# The Impact of Motivation on Sustained Attention in Very Preterm and Term-born Children: An ERP Study

**DOI:** 10.1177/10870547251313888

**Published:** 2025-01-28

**Authors:** Jenny Retzler, Madeleine J. Groom, Samantha Johnson, Lucy Cragg

**Affiliations:** 1 School of Psychology, University of Nottingham, UK; 2 Centre for Cognition and Neuroscience, School of Human and Health Sciences, University of Huddersfield, UK; 3 Division of Psychiatry and Applied Psychology, Institute of Mental Health, School of Medicine, University of Nottingham, UK; 4 Department of Population Health Sciences, University of Leicester, UK

**Keywords:** attention, very preterm, motivation, arousal

## Abstract

**Objective:**

To compare the effect of motivational features on sustained attention in children born very preterm and at term.

**Method:**

EEG was recorded while 34 8-to-11-year-old children born very preterm and 34 term-born peers completed two variants of a cued continuous performance task (CPT-AX); a standard CPT-AX with basic shape stimuli, and structurally similar *motivating* variant, with a storyline, familiar characters, and feedback.

**Results:**

Higher hit rates, quicker response times and larger event-related potential (ERP) amplitudes were observed during the motivating, compared with the standard, task. Although groups did not differ in task performance, between-task differences in ERPs associated with orienting were larger in term-born than very preterm children.

**Conclusion:**

The findings add to previous evidence of disruption to the brain networks that support salience detection and selective attention in children born preterm. Manipulations that increase intrinsic motivation can promote sustained attention in both term-born and very preterm children.

## Background

Attention difficulties are among the most common adverse neurobehavioral outcomes for children born very preterm (<32 weeks of gestation; Ask et al., 2018; Brogan et al., 2014; Larsen et al., 2024). While there is a consensus that the neurodevelopmental condition Attention-Deficit/Hyperactivity Disorder (ADHD) usually results from gene-environment interactions (Faraone & Larsson, 2019), evidence in 8-year-olds born at 33 weeks’ gestation or earlier (Ask et al., 2018) and adolescents born preterm (<37 weeks’ gestation; James et al., 2020) suggests that familial factors (genetic or environmental) do not explain the development of inattention in preterm populations. Instead, it is proposed that, in these children, the causative pathway for inattention results from altered brain development following birth at a gestation where the brain is immature. Indeed, measures of neonatal cerebral development have been associated with persistent inattention/hyperactivity problems in later childhood (Bora et al., 2014; Rogers et al., 2018). Questions, therefore, arise regarding whether the neurocognitive basis of inattention is equivalent in term-born and very preterm groups. Below, we discuss how theories and evidence of associations between attention, arousal regulation and motivation, considerations of the nature of motivation, and growing evidence of atypical arousal regulation within very preterm samples, have informed this study.

### Attention, Arousal Regulation, and Motivation

Theories of ADHD have proposed that differences in the regulation of arousal may contribute to problems sustaining attention (e.g., Cognitive-Energetic Model, Sergeant, 2005; Dynamic Developmental Theory, Savgolden et al., 2005). Broadly speaking, the term “arousal” is used to describe the state of sensory alertness experienced by an individual. Importantly, it is considered dynamic (Mayes, 2000), changing between and within individuals and fluctuating in response to external and internal factors. In relation to sensory input, it is often conceptualized as a phasic physiological response—or the “what is it” reaction (van der Meere et al., 2010). A systematic review found evidence to support the role of arousal in sustaining attention, with relatively consistent findings of hypoarousal of the autonomic nervous system in participants with ADHD, both at rest and during tasks requiring cognitive control or sustained attention (Bellato et al., 2020).

Motivation is thought to affect arousal regulation; a core assumption of the State-Regulation Model of ADHD is that the “effort system” (or motivation system) controls arousal regulation (van der Meere et al., 2010), such that when motivation is high, individuals who may not be at the optimal level of arousal to respond to task demands are able to allocate additional physiological resource (effort). This interaction between motivation and arousal regulation may explain why so much context variability is observed across studies of sustained attention in individuals with ADHD (Champ et al., 2023), as well as seemingly paradoxical observations of the hyper-focusing phenomenon (an extended state of sustained focused attention on an activity, often to the exclusion of all else) in those with ADHD (Groen et al., 2020).

Empirical evidence demonstrates manipulations of motivation can affect processing in individuals with and without ADHD. For example, Groom et al. (2010) found that introducing points-based incentives on a go/no-go task increased enjoyment of the task and improved performance for participants with and without ADHD. Moreover, electrophysiological event-related potential (ERP) markers of task-relevant attention (P3) and inhibitory control (N2) were enhanced by the introduction of incentives in both groups. Patterns indicated that when incentivized, the P3 and N2 amplitudes observed in the ADHD group were closer to those observed in the non-ADHD controls in the non-incentive baseline condition. Later work (Groom et al., 2013) indicated that incentives also enhanced ERP markers of performance monitoring (ERN and Pe) in the ADHD group only. These effects were similar to, although smaller than, those produced by methylphenidate medication.

However, because experimental studies assessing how manipulations of motivation affect those with ADHD have been heavily focused on differences in reward processing, they primarily assess the impact of external reinforcers, such as points, tokens or money, as motivators. It remains to be seen whether there are any effects of manipulations to increase motivation in a more intrinsic manner (Morsink et al., 2022). Below we outline why different types of motivation may impact arousal regulation differently, and how increasing understanding of this nuance is crucial to characterizing differences in arousal regulation and the subsequent development of effective approaches to support attention.

### The Importance of the Nature of Motivation

Given the theoretical importance of the role of motivation in arousal regulation in those with attentional difficulties, researchers have more recently begun considering how applying the self-determination theory (SDT; Deci & Ryan, 2008) to ADHD may advance understanding and management of inattention (Champ et al., 2023; Morsink et al., 2022; Rogers & Tannock, 2013). It should be noted that our study was not designed *a priori*, to test the SDT, but it is a theoretical framework that is helpful for understanding why and how the nature of motivation is important in attention regulation.

Briefly, the SDT considers how far situations support a person’s satisfaction of the basic psychological needs of autonomy, competence and relatedness. The extent to which these needs are satisfied will determine the level of motivation, which, in turn, corresponds to the manner by which someone can regulate their behavior, and thus can impact task-engagement and performance. There are four levels of motivation. The least optimal level of motivation is *amotivation*, which results in no regulation. Next, is *controlled motivation*, which activates external or introjected regulation, reflecting situations where someone is motivated to complete a task mainly through incentives or to please others. Better still is *autonomous motivation*, which activates identified or integrated regulation, where someone is motivated because the task aligns to some extent with their internal values. The most optimal level is *intrinsic motivation*, which activates intrinsic regulation, where the task is completed purely because it is enjoyable and satisfying and the individual can self-regulate effortlessly.

According to this perspective, external incentives as used in prior studies of individuals with ADHD, are unlikely to be sufficient to introduce intrinsic motivation; in fact, rewards, punishments or other extrinsic motivators may even reduce the sense of volition and be experienced as controlling (Champ et al., 2023; Morsink et al., 2022). Instead, according to SDT, intrinsic motivation requires an individual to find a task or activity enjoyable, interesting and satisfying (Di Domenico & Ryan, 2017). The authors propose a “neurobiocognitive” explanation, whereby intrinsic motivation is supported by dopaminergic activity, which stimulates dynamic switching between the neural networks supporting salience detection and attentional control. The overlap between these neural substrates and those implicated in ADHD is extensive, and is detailed comprehensively in Champ et al. (2023).

Such theories are informed by our growing understanding of the neurobiology of salience detection and how it corresponds to arousal regulation. Building on early evidence of a right-lateralized ventral fronto-parietal network that orients attention to behaviorally relevant or unexpected salient stimuli and maintains vigilance (Corbetta & Shulman, 2002), subsequent research led to proposals of a “salience network” (SN; Uddin, 2015). This is thought to drive the switch from the default mode network (DMN), which is typically active at rest or when mind-wandering, to the central executive network (CEN), which is important for controlled attention and goal directed action (Goulden et al., 2014; Sridharan et al., 2008). Neuroimaging evidence supports the notion that motivational factors can “normalize” the level of activity in these networks for those with ADHD. Liddle et al. (2011) found children with ADHD withdrawn from methylphenidate showed less task-related DMN deactivation than non-ADHD controls in a low incentive condition, indicative of poor arousal regulation, but there was no difference between groups when motivational incentives were introduced. The authors proposed that in ADHD, effective DMN deactivation may only occur when motivational salience is high. However, it should be noted that these studies used external reinforcers and the impact of manipulations to intrinsic motivation on these processes is less well understood in ADHD.

### Arousal Regulation and Very Preterm Birth

Findings that those born very preterm are susceptible to frequent lapses in attention (de Kieviet et al., 2012) has prompted suggestions that arousal regulation may also be atypical in this population. Empirical studies have since provided emerging support for this idea. Jaeger et al. (2019) found that even though “low-risk” preterm-born (28 to 36 weeks’ gestation) 5-to-6-year-olds with no observable behavioral difficulties (including no elevation of ADHD symptoms) performed as well as term-born peers on an oddball task, oddball-evoked increases in the P3 ERP were absent in the preterm children, which they interpreted as reflecting poor arousal regulation. A subsequent study of the same sample reported that the preterm-born children displayed slower decision time and reaction time relative to term-born peers only in an uncued response time task, and not in a cued response time task (Jaeger et al., 2021). The authors concluded that regulation of tonic alertness, which reflects intrinsic and long-term regulation of attentional arousal, may be affected by birth at preterm gestations, while phasic (reactive) alertness may not be. Moreover, they indicated that the presence of cues may promote better task performance.

Evidence of disrupted arousal regulation has also been observed in a sample of preterm-born adolescents recruited from mainstream education, which did include those with attentional difficulties. James et al. (2018) compared preterm-born (<37 weeks’ gestation) adolescents with groups of term-born peers with and without ADHD. Those born preterm showed similar performance to the term-ADHD group in the speed and variability of response time, and ERPs reflecting response preparation, each of which related to ADHD symptomatology in those born preterm. Of particular interest, however, was the finding that when manipulations of event rate and incentives were introduced to tasks, adolescents born preterm demonstrated less malleability in attention allocation and arousal (measured by the P3 ERP and skin conductance) than their term-born peers. This pattern was not observed in the ADHD group, indicative of unique effects of preterm birth on arousal regulation in response to experimental manipulations of motivational state. However, the samples in both James et al. (2018) and Jaeger et al (2019; 2021) included children born at moderate-to-late preterm gestations (32 to 37 weeks) and thus may not reflect the extent to which arousal regulation is affected in children born very preterm. To our knowledge, no studies have investigated the impact of manipulations of motivation, nor ERP measures of attention, in children born very preterm in the mid-childhood age range.

### The Current Study

The current study sought to investigate the impact of manipulations to increase intrinsic motivation on sustained attention and neural regulation of attentional resource, measured using ERPs, in very preterm and term-born children. To date, studies have been limited in the extent to which they have been able to address whether the neurocognitive basis of inattentive behavior is equivalent in term-born and very preterm groups, primarily recruiting term-born “control” groups who are unlikely to show the full range of attentional difficulties. This approach may not accurately capture associations between cognition and (in)attention in term-born children. To our knowledge, only one research team (James et al., 2018) has compared those born preterm (<37 weeks’ gestation) to a term-born comparison group with ADHD diagnoses, the latter of which were recruited from a sample who had participated in other studies of adolescents with ADHD. Their analyses compared adolescents born at any preterm gestation to adolescents born at term with and without ADHD. In contrast, the present PATCH (Preterm birth and ATtention in CHildren) Study, focused on middle-childhood and those born at very preterm gestations. Rather than grouping based on ADHD status, we instead, aimed to recruit a term-born sample of children with a wide distribution of attention levels, to better mirror the distribution observed in very preterm samples and facilitate between-group comparisons of the neurocognitive mechanisms underlying inattention.

The PATCH Study used adapted variations of a cued-continuous performance task (cued-CPT; CPT-AX). CPTs are known to be sensitive to the behavioral differences observed between children in the general population and those with ADHD (Huang-Pollock et al., 2012) and born very preterm (Mulder et al., 2009). Studies have shown that CPT task performance is better predicted by inattentive symptoms than hyperactive-impulsive symptoms (Chhabildas et al., 2001). While CPTs generally involve detection of an infrequent target letter (X) among a sequence of distractor letters, in the cued-CPT participants respond to infrequent cue-target (A-X) sequences among distractor stimuli. This requires maintenance of attention throughout long periods of non-response, in order to correctly respond when the rare cue-target sequence is presented. Moreover, presentation of the X “target” stimulus sometimes occurs without a preceding cue (henceforth referred to as an “uncued target,” or B-X), and thus the task requires higher order decision-making and use of memory while evaluating the task-relevance of the stimulus.

Moreover, cued-CPTs are known to evoke ERP components that have been linked to neural substrates of attention (Riccio et al., 2002). ERPs were assessed in the current study to measure the impact of task manipulations on neural processing, and to facilitate detection of neural effects even where task performance is unaffected (Jaeger et al., 2019). Specifically, the study investigated the P1, P2, P3, and CNV components.

P1 is one of the earliest components that can be modulated by attention, and larger amplitudes are interpreted as greater amplification of a task-relevant stimulus (Hillyard et al., 1998). While some studies show atypical P1 responses in ADHD groups relative to controls, this is not a consistent finding (Kaiser et al., 2020). Few studies report examination of the P1 in very preterm samples (only Hövel et al., 2014; Mikkola et al., 2007), and no studies report examination of this component in middle childhood. Taken together with evidence from studies of other preterm samples, such as one including moderately preterm-born infants (23 to 34 weeks’ gestation; Suppiej et al., 2015) and another with older children in a very low birth weight sample (which included many children born at very preterm or earlier gestations; Potgieter et al., 2003), findings indicate that P1 amplitudes are sometimes, but not always, reduced in preterm relative to term-born children.

P2 is another component where amplitudes increase with attention, and it is thought to reflect the perceptual matching of the stimulus presented to our internal representations (Luck & Hillyard, 1994), in order to terminate sensory processing and trigger subsequent processes (Hansen & Hillyard, 1988; Oades, 1998). While differences in the P2 component have been associated with ADHD status, the direction of effects is inconsistent (Kaiser et al., 2020). To our knowledge, only Hodel et al. (2019) and Lavoie et al. (1998) report examination of the P2 component in relation to preterm birth, both finding no P2 differences when comparing extremely preterm (<28 weeks’ gestation) 5-year-olds, and moderate-to-late preterm (32 to 36 weeks’ gestation) 4- and 5-year-olds, respectively, to their term-born peers. However, due to the links with ADHD and scarcity of research in preterm populations, we felt this was an important component to examine.

P3 is a component thought to be associated with evaluation of the task-relevance of the stimulus. It has been closely linked to autonomic arousal (Nieuwenhuis et al., 2011) and larger amplitudes tend to occur when individuals are paying more attention. Evidence consistently shows reduced P3 amplitude in ADHD groups relative to non-ADHD controls, and it has been proposed that this component is the most sensitive biomarker for ADHD (Kaiser et al., 2020). Moreover, studies have observed reduced amplitude of P3 in preterm-born adolescents compared with term-born adolescents with and without ADHD (Rommel et al., 2017). However, in Potgieter et al.’s (2003) study, a typicalities in P3 were attributed to ADHD status rather than birth weight status, and Mikkola et al. (2007) found no between-group difference in the P3 component between 5-year-olds born very preterm and their peers. In response to some tasks, the P3 can be separated into two components; the earlier component, P3a, is thought to reflect evaluation of importance, while the later component, P3b, may reflect the updating of working memory (Polich, 2007), but much of the research linking the P3 to inattention does not differentiate between earlier and later components.

Contingent negative variation (CNV), is a negative deflection that follows cue presentation and precedes an anticipated “go” target. It is thought to reflect stimulus expectancy and motor response preparation, whereby larger amplitude CNVs are associated with faster response times (Wright et al., 1995). Amplitudes tend to be smaller in ADHD populations (Kaiser et al., 2020) and relate inversely to symptoms (Ortega et al., 2013). Rommel et al. (2017) found attenuated CNV amplitudes in both preterm-born and term-born adolescents with an ADHD diagnosis relative to those born at term without ADHD, and reduced amplitude CNV was also observed in preterm-born (24 to 36 weeks’ gestation) 6-to-11-year-olds relative to term-born peers (Mento et al., 2022).

We hypothesized we would observe increased attention (as indexed by ERPs) and improved task performance in the motivating task condition compared with the standard task. Because the task involves orienting of attention in response to cues, it was anticipated that cue-evoked ERP effects may reflect activation of the right-lateralized ventral network (Corbetta & Shulman, 2002), thus we investigated the hemispheric location of task-related differences, expecting effects to be right-lateralized. Based on previous research, we predicted impaired performance and reduced ERP amplitudes in the very preterm group, compared with the term-born group. We further explored the effect of manipulating intrinsic motivation (task type) on performance and ERPs, predicting that this may improve attention in the very preterm group more than the term-born group (group*task interaction), or that these effects would be equivalent across groups (main effect of task type).

## Methods

### Sample

Recruitment is described in full in Retzler et al. (2019), and the supplementary material provides detail on participant flow in Supplemental Figure S1. Briefly, 65 8-to-11-year-olds born very preterm (≤32 weeks’ gestation) participated in the PATCH study after invitations were sent to parents of eligible children admitted to neonatal intensive care units in the Nottingham University Hospitals NHS Trust. Term-born children from the local community were invited to take part in an initial screening stage that used the parent-completed Strengths and Weaknesses of ADHD and Normal Behaviors scale (Arnett et al., 2013; Swanson et al., 2012) to ensure the full range of attention (far below average, below average, slightly below average, average, slightly above average, above average, far above average) was captured across the term-born group. Of those invited to take part in the full study, 48 8-to-11-year-olds born at term (≥37 weeks gestation) participated. No children who participated in the study were on stimulant medication for ADHD at the time of participation. The analysis presented here includes all 34 children from each group for whom key data were available; specifically, those with task and EEG data for both CPT-AX tasks.

### Data Collection

#### Measures

Parent-rated inattention was measured using the Strengths and Weaknesses of ADHD and Normal-behavior (SWAN; Swanson et al., 2012). Further assessments to characterize the sample more fully included age-standardized estimates of full scale IQ (FSIQ) derived from the vocabulary and matrices subtests from the Wechsler Abbreviated Scale for Intelligence—Second Edition (WASI-II; Wechsler, 2018); ADHD symptoms and risk of ADHD using the Conners 3-P (Conners, 2008); socio-economic status tertile based on the postcode ranking according the English Indices of Multiple Deprivation (McLennan et al., 2011); and bespoke items in a parent questionnaire reporting the child’s age and ethnic background.

**Figure 1.**
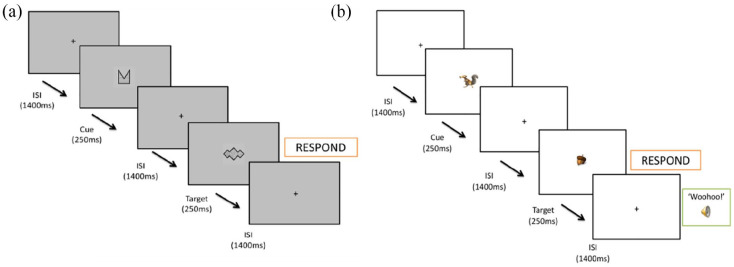
Schematic of cue-target (A-X) sequence for the standard (a) and motivating (b) tasks.

Sustained attention was tested using two variants of a cued continuous performance task (CPT-AX) programmed using PsychoPy software (Peirce et al., 2019). Both variants included 4 blocks of 100 trials in which stimuli were presented in the centre of the screen in a continuous stream. Each stimulus was preceded by a central fixation cross for 1400 ms, with the stimuli presented for 250 ms (see Figure 1). There were 11 irrelevant distractor stimuli (“Y”), a cue stimulus (“A”) and a target stimulus (“X”). Children were instructed to respond by pressing a button as soon as possible whenever they saw a cue-target (A-X) trial sequence. A-X “go” sequences occurred on 10% trials per block. All remaining trials were “no-go” trials, including those where A and X stimuli were presented out of sequence (B-X or A-Y). This provided the following trial types for analysis: 50% irrelevant distractor and non-target stimulus sequences (B-Y), 20% trials in which the cue was the stimulus present (A), 10% trials which presented an irrelevant (non-target) distractor stimulus that was preceded by the cue (A-Y), and 10% trials which presented the stimulus designated the “target” but preceded by an irrelevant distractor stimulus (not the cue; B-X). A correct hit was defined as an A-X trial in which a response was made between 200 and 1650 ms after the cued target was presented. For each child, and in each task, hit rate (go-accuracy), median RT (speed) and standard deviation of RT (response time variability) were computed from “go” trial types. Commission errors were calculated as the number of incorrect responses on “no-go” trials.

The two tasks were programmed to have the same presentation structures with the same order of the trial types (although the stimuli themselves differed; Supplemental Figure S2 depicts the stimuli used in each task), and the same response requirements. Within each task the shapes designated as the “cue” and “target,” and order of presentation of all distractor stimuli were the same across all children. While the standard task used abstract shapes and monochrome colors, the motivating task variant used colorful characters from the popular “Ice Age” television and film series; it was thought that this would make the task features more salient. While the standard task gave clear instructions of task requirements without a rationale, the motivating task variant had an additional storyline that added rationale for the response requirements and linked these back to a common theme in the “Ice Age” series - helping Scrat the squirrel (the cue; A), to collect acorns (the target; X). This was intended to provide enjoyment and interest, thus generating intrinsic motivation despite the lengthy task. Finally, while the standard task did not provide participants with any feedback, in the motivating task an audio “woohoo” was played through headphones immediately following a correct “go” response, or an “oops” for an error of omission on go trials. In neither task were any external rewards or punishments provided, but it is thought that immediate feedback may have fulfilled basic needs of affirming “competency,” thus also increasing the potential for intrinsic motivation.

#### Procedure

These data were recorded as part of a wider study where children first completed a battery of cognitive tests (approx. 45 min), with breaks, before having a longer break and the EEG set-up. The EEG set-up took approximately 45 min, and children were entertained with an age-appropriate film of their choosing (not including the film used in the motivating task variant).

CPT-AX completion took place in a windowless shielded room. Children were seated a comfortable distance from the screen, wearing headphones and accompanied by a researcher, who read the task instructions aloud and remained present to ensure the child felt comfortable and understood task requirements. Children completed each of the tasks in turn, with the order of the tasks counterbalanced across participants. Each task took approximately 15 min to complete, and children could take breaks between each block of 100 trials, as well as between the two tasks. Responses were recorded using a Cedrus RB730 button box, and a star-shaped sticker was placed on the response key (left-most button) as a reminder. Children were instructed to use their right hand and to keep their finger positioned above the response button to allow them to respond as quickly as possible.

#### EEG Recording

The EEG was recorded at a 1000Hz sampling rate, using a DBPA-1 Sensorium bioamplifier (Sensorium Inc., Charlotte, VT). Voltage was recorded from 117 active silver/silver-chloride (Ag/AgCl) scalp electrodes using caps customized for our lab (easycap, Munich, Germany) with twisted and fixed electrode cables. We used different caps to account for different head sizes (50, 52, 54, 56, 58 cm). Electrode positions were based upon the 10/5 system, an extension of the traditional 10/20 system (Oostenveld & Praamstra, 2001), at 117 sites (see Supplementary Materials for more details). An electrode on the left mastoid served as the recording reference and the ground electrode was placed on the chin. Two additional electrodes were placed by the outer canthi of each eye (LHE and RHE) to measure horizontal eye movements, while a further electrode was placed below the left eye (LIO) to measure vertical eye movements. In line with manufacturer recommendations, electrode impedances were brought below 50kΩ prior to task completion.

### Analysis

#### EEG Pre-processing

EEG data were analyzed offline using MATLAB with purpose-written scripts which used EEGlab (Delorme & Makeig, 2004), ERPlab (Lopez-Calderon & Luck, 2014) and the Fully Automated Statistical Thresholding for EEG Artefact Rejection (FASTER; Nolan et al., 2010) plug-ins. Data were filtered with notch filters at 50 and 100 Hz and a bandpass filter between 0.5 and 100 Hz, down-sampled to 500 Hz, and average referenced. Epochs were defined as windows from −200 to 1650 ms and low pass filtering was applied at 40 Hz. Artefact rejection, conducted using FASTER (Nolan et al., 2010), assessed the EEG data at four levels; channels, epochs, independent components, and single-channel single-epochs, to identify contaminated data (z score of ±3 for that metric; full details provided in Supplementary Materials).

Following rejection procedures, the average number of trials per participant for cue (A) ERPs was 79.88 (*SD* = 1.47; 99.85% trials retained; motivating task *M* = 79.75, *SD* = 1.47, 99.69% trials retained; standard task *M* = 80.00, *SD* = 0.00, 100% trials retained), for irrelevant distractor (B-Y) ERPs was 178.12 (*SD* = 4.73; 89.06% trials retained; motivating task *M* = 177.99, *SD* = 4.73, 88.99% trials retained; standard task *M* = 178.25, *SD* = 2.88, 89.13% trials retained) and for cued-target (A-X) ERPs was 39.93 (*SD* = 0.87; 99.83% trials retained; motivating task *M* = 39.85, *SD* = 0.87, 99.63% trials retained; standard task *M* = 40.00, *SD* = 0.00, 100% trials retained). Average number of trials per ERP average did not differ between groups or between tasks for any trial type (*p* > .1).

#### ERP Computation

To maximize the signal-noise ratio and facilitate topographical comparisons, EEG signal was averaged across electrodes within clusters that reflected 12 areas of the scalp in terms of coronal (frontal, central, parietal, occipital) and sagittal (left, midline, right) position (see Supplementary Material for details).

**Figure 2.**
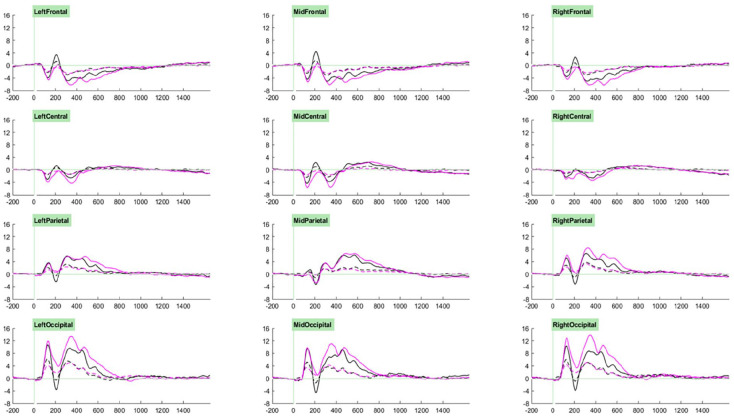
Grand averages of cue (solid) and irrelevant distractor (dashed) trials for the standard (black) and motivating (pink) task variants.

**Figure 3.**
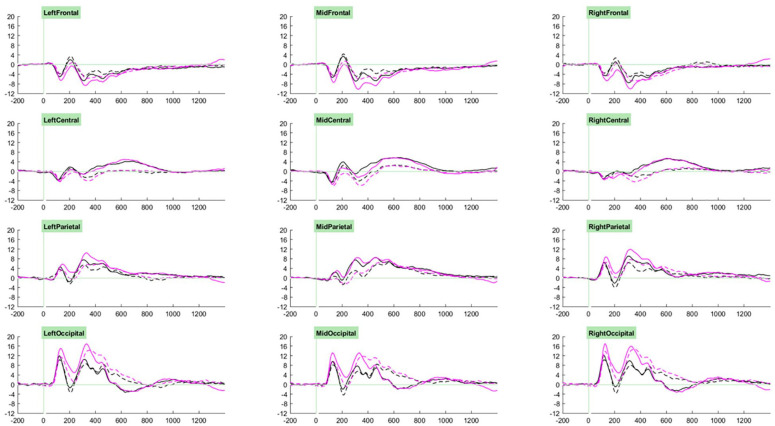
Grand averages of cued targets (solid) and uncued targets (dashed) trials for the standard (black) and motivating (pink) task variants.

Our ERP analysis took a whole-waveform approach, where we investigated group- and task- related effects for each component. Selection of the cluster and time windows in which to conduct analyses for each component was guided through visual examination of the grand average ERPs associated with trial types of interest across participants (see Figures 2 and 3). Accordingly, analyses for P1 components were measured at parieto-occipital clusters between 70 and 200 ms, those for P2 components were measured at fronto-central clusters between 170 and 250 ms, those for P3a components were measured at parieto-occipital clusters between 250 and 400 ms, and those for P3b components were measured at parieto-occipital clusters between 400 and 600 ms. Cue-CNV amplitudes were initially measured at centro-parietal clusters between 1200 and 1650 ms, however on account of the very small amplitudes observed (likely due to the low proportion of “go” trials) and lack of effects of interest, these results are not reported.

To facilitate between-task comparison of attention-related processing and reduce the contribution of visual properties that were common within tasks (e.g., background luminance) to the ERP analysis, difference waves were computed (Kappenman et al., 2021) and are denoted by the subscript “diff” suffix. Because we were interested in explaining the extent to which differences in attention were affected by task manipulations and whether this difference changed dependent on group, it was felt that excluding incorrect trials (omission/commission errors) would exclude trials where attentional modulation was not effective, thus computations used ERP data from all available trials for each trial type, not just correct trials. Accordingly, for each participant on each task, difference waves were computed that reflected the differences in orienting between cue trials and irrelevant distractor trials (A minus B-Y), and differences in target processing between cued target “go” trials and uncued target “no-go” trials (A-X minus B-X). Mean amplitude measures were used as the measure of electrical activity to avoid issues with peak detection caused by the impact of the difference wave computation on peak distributions, as well as the sensitivity of peak-based measures to noise and individual differences in latency, inter-trial variability and trial numbers.

#### Statistical Analysis

Mixed analyses of variance (ANOVA) were used to assess the impact of Group (between-subjects) and Task (within-subjects) on each task performance metric. For ERP analyses, an additional within-subjects factor of topographical Cluster was modelled and significant 3-way interactions were followed up with mixed 2 (Group) x 2 (Task) ANOVAs at each electrode cluster, with Sidak-corrected post-hoc comparisons. To protect against a Type II error on *a priori* effects of most interest, a threshold of *p* < .05 was applied to Group- and Task- related effects or interactions, while a more conservative significance threshold of *p* < .01 was applied to all other effects.

Because those born very preterm were significantly older than those born at term, we followed procedures for including covariates recommended in Schneider et al. (2015). ANOVAs were performed both with and without the addition of the covariate of Age, which was mean-centred. For most analyses, the effect of Age and interactions with Age did not reach the threshold for significance, thus effects were reported from the ANOVA. For comparisons of RT and RT variability, a significant effect of Age was observed. As such, between-subjects effects and those involving interactions with Age were reported from the ANCOVA, while within-subjects effects and interactions that did not involve Age were reported from the ANOVA; degrees of freedom may vary as a result.

For variables where Levene’s test indicated the assumption of homogeneity of variance was violated, variance was examined. Given group sizes were equal, provided the largest variance was no more than 9 times the smallest variance, it was assumed the ANOVA remained robust to these violations (Keppel et al., 1992). Greenhouse-Geisser corrections have been applied where Mauchley’s test indicated violations to the assumption of sphericity. Pearson’s correlations between task performance and ERP metrics were also conducted to support interpretation of ERP components, and can be found in Supplementary Materials.

## Results

### Sample Characteristics

A full comparison of sample characteristics between very preterm and term-born children included in this ERP analysis are provided in the Supplementary Material (Supplemental Table S1), with key features summarized in Table 1 below. Compared with term-born children, those born very preterm were significantly older (*p* = .004) and had significantly lower IQ (VP: *M* = 103.50 points, *SD* = 12.74 points; Term: *M* = 112.64, *SD* = 8.74 points; *p* = .001), but were well-matched on all other variables, including both SWAN and Conner’s 3-P parent-rated inattention and hyperactivity, and the proportion scoring above clinical cut-offs for ADHD (see Table 1 and Supplemental Table S1).

**Table 1.** Characteristics of Term-born and Very Preterm Children.

Participant demographics	Very preterm (*n* = 34)	Term (*n* = 34)	*p*
Gestational age at birth (weeks)^a^; Mean (*SD*)	30^+1^ (1^+6^)	40^+0^ (1^+1^)	—
Birth weight (kg); Mean (*SD*)	1.53 (0.46)	*Not available*	—
Age at assessment (years); Mean (*SD*)	10.04 (0.92)	9.54 (1.04)	0.040*
Sex; *n*(%) female	16 (47.1%)	16 (47.1%)	>.999 *n.s.*
Conner’s 3 scores above clinical cut offs for DSM ADHD/I; *n*(%)	9 (26.5%)	7 (20.6%)	.567 *n.s.*
Conner’s 3 scores above clinical cut offs for DSM ADHD/C; *n*(%)	9 (26.5%)	8 (23.5%)	.779 *n.s.*

*Note. SD* = standard deviation.

a 2 children in the VP sample were born at gestations of fewer than 28 weeks, meeting criteria for extremely preterm birth.

* *p* < .05, *n.s.* = not significant.

### Task Performance

Overall, task performance was better for the motivating task than the standard task.

Significant main effects of Task were observed for hits (accurate responses on go trials; *F* [1, 66] = 4.61, *p* = .036, η_*p*_^2^ = .07), and RT (*F* [1, 65] = 55.97, *p* < .001, η_*p*_^2^ = .46), with greater accuracy and faster RT on go trials for the motivating task than the standard task (see Table 2; histograms of the distribution of RT on each task are presented in Supplemental Figure S3). No significant effects of Task were found for RT variability (*F* [1, 65] = 1.54, *p* = .219, η_*p*_^2^ = .02) or commission errors (go responses on no-go trials; *F* [1, 66] = 0.32, *p* = .576, η_*p*_^2^ < .01).

**Table 2.** Marginal Means and Standard Error of the Mean for Task Performance Metrics Split by Group and Task.

	Very preterm (*n* = 34)		Term (*n* = 34)	
	Motivating	Standard	Motivating	Standard
Hits ^a^ (mean, SEM)	37.35 (0.64)	36.74 (0.83)	36.38 (0.64)	35.24 (0.83)
Response time ^b^ (mean, SEM)	379.85 (12.19)	431.49 (12.86)	411.95 (12.19)	461.04 (12.86)
Response time variability ^b^ (mean, SEM)	145.27 (12.16)	153.90 (10.75)	145.97 (12.16)	160.39 (10.75)
Commission errors (mean, SEM)	8.76 (2.52)	7.03 (1.54)	7.88 (2.52)	7.68 (1.54)

*Note.* ^a^The maximum number of hits possible was 40. ^b^Means and standard error for these variables control for age.

There was a significant main effect of Age on RT (*F* [1, 65] = 11.76, *p* < .001, η_p_^2^ = .15), and RT variability (*F* [1, 65] = 8.49, *p* = .005, η_p_^2^ = .12), but not on hits (*F* [1, 65] = 3.50, *p* = .066, η_p_^2^ = .05) or commission errors (*F* [1, 65] = 1.42, *p* = .238, η_p_^2^ = .02). Age did not interact with Task in any comparison (*p*s > .05).

There were also no significant effects of Group on task performance (hits *F* [1, 66] = 1.63, *p* = .207, η_p_^2^ = .02; RT *F* [1,65] = 3.45, *p* = .068, η_p_^2^ =.05; RT variability *F* [1, 65] = 0.07, *p* = .790, η_p_^2^ < .01; commission errors *F* [1, 66] < 0.01, *p* = .961, η_p_^2^ < .01), and no significant interactions between Task and Group (hits *F* [1, 66] = 0.42, *p* = .522, η_p_^2^ < .01; RT *F* [1, 65] = 0.03, *p* = .855, η_p_^2^ < .01; RT variability *F* [1, 65] = 0.09, *p* = .764, η_p_^2^ < .01; commission errors *F* [1, 66] = 0.20, *p* = .659, η_p_^2^ < .01).

### Event-related potentials

Table 3 summarizes the ERP findings. Components reflecting orienting differences showed both Group- and Task- related effects and are reported in full below. For target-evoked components, although some significant Task-related effects were observed, the effects of Group, and its interaction with Task, were minimal, and findings are reported in the Supplementary Materials. For all components the main effect of the covariate of Age, and interactions between Age and Task, Age and Cluster, and between Age, Task and Cluster did not meet the threshold for significance (*p*s > .01), thus all findings are reported from the ANOVA without the covariate. Exploratory correlational analyses of the links between ERP amplitudes and task performance are also reported in Supplementary Materials.

**Table 3.** Summary of ERP Results.

Component	Main effects of task	Main effects of group	Topographic distribution	Interaction effects
Cue minus distractor ERP waveforms				
Orienting-P1_diff_	Motivating > standard	None	Bilateral occipital	Group * Task * Cluster interaction *In the LO and RO clusters Term > VP in the Motivating task only, & Motivating > Standard in Term children only*
Orienting-P2_diff_	Standard > motivating	None	Mid-frontal	Task * Cluster interaction *Task-related differences in right and midline fronto-central clusters were bigger than those in left fronto-central clusters*
Orienting-P3a_diff_	Motivating > standard	None	Bilateral occipital	Group * Task * Cluster interaction *In the LO and RO clusters, Term> VP in the Motivating task only, & task-related effects were stronger, or only present in term children*
Orienting-P3b_diff_	Motivating > standard	Term > VP	Right occipital	Task * Cluster interaction *Task-related effects were stronger in the RP and RO clusters relative to left or midline clusters*
Cued target minus uncued target waveforms				
Target-P1_diff_	Motivating > standard	None	None	None
Target-P2_diff_	None	None	Mid-central	Task * Cluster *Motivating > Standard in RF cluster*
Target-P3a_diff_	None	None	Mid-parietal	Task * Cluster *Motivating > Standard only in LP and RP clusters* Group * Cluster *In Term-born children, right lateralisation, but in VP children mid-parietal distribution*
Target-P3b_diff_	None	None	Occipital	Task * Cluster *Motivating > Standard only in occipital clusters*

*Note.* Suffix of _diff_ denotes difference wave.

#### Orienting-P1_diff_ (Cue Minus Distractor)

**Figure 4.**
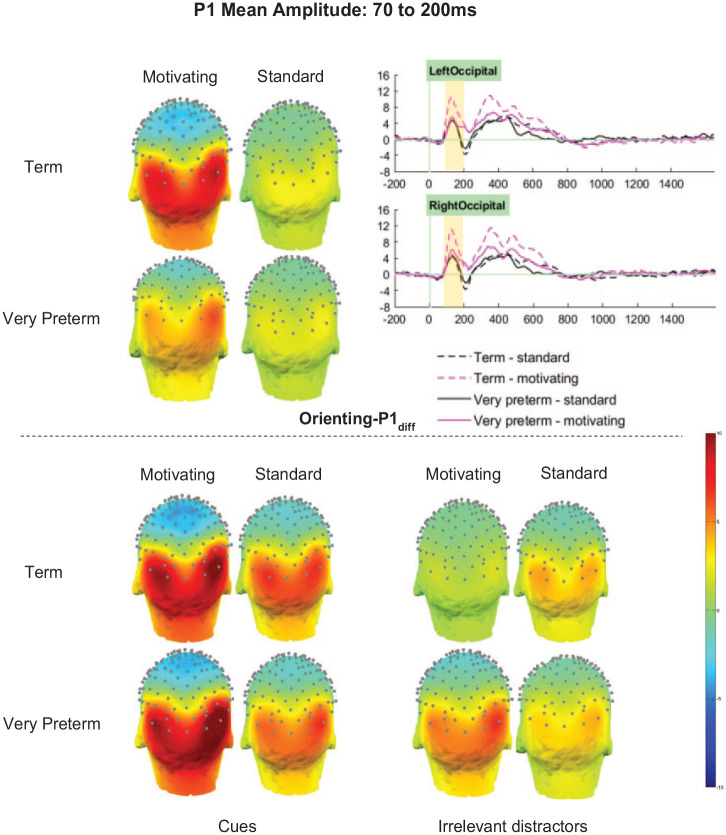
Orienting-P1 mean amplitude between 70 and 200 ms.

For the Orienting-P1_diff_ there was no significant main effect of Group (*F* [1,66] = 1.61, *p* = .209, η_p_^2^ < .02). However, there were significant main effects of Task (*F* [1, 66] = 11.07, *p* = .001, η_p_^2^ = .14) and Cluster (*F* [2.90, 191.07] = 80.78, *p*.001, η_p_^2^ = .55), and significant Group by Task (*F* [1, 66] = 4.53, *p* = .037, η_p_^2^ = .06), Group by Cluster (*F* [2.90, 191.07] = 5.86, *p*.001, η_p_^2^ = .08), and Task by Cluster (*F* [2.52, 166.03] = 23.54, *p*.001, η_p_^2^ = .26) interactions. These were qualified by a significant three-way interaction between Group, Task and Cluster (*F* [2.52, 166.03] = 6.19, *p*.001, η_p_^2^ = .09), which indicated that the Task-related differences in Orienting-P1_diff_ amplitude differed both by Group and by Cluster (see Figure 4).

Orienting-P1_diff_ amplitudes in the left-parietal cluster were unaffected by Task or Group (*p*s > .1). In the midline and right parietal clusters, amplitudes were significantly higher for the motivating task than the standard task (MP *p* = .032, η_p_^2^ = .07; RP *p* = .023, η_p_^2^ = .08; MO *p* = .004, η_p_^2^ = .12), but there were no significant effects of, or interactions with, Group. Significant effects of Group (*p* = .020, η_p_^2^ = .08) and Task (*p*.001, η_p_^2^ = .03) in the left-occipital cluster, and of Task only in the right occipital cluster (*p*.001, η_p_^2^ = .26), were qualified by significant interactions between Task and Group (LO *p* = .003, η_p_^2^ = .13; RO *p* = .015, η_p_^2^ = .09).

Sidak-corrected pairwise comparisons showed that in both the LO and RO clusters, greater Orienting-P1_diff_ amplitudes in term-born relative to very preterm children were observed only in the motivating task (LO motivating *p* = . 003, η_p_^2^ = .13; LO standard *p* = .794, η_p_^2^ < .01; RO motivating *p* = .021, η_p_^2^ = .08; RO standard *p* = .592, η_p_^2^ < .01). Moreover, in both clusters, significantly greater amplitudes for the motivating task relative to the standard task were observed only in those born at term (LO *p*.001, η_p_^2^ < .33; RO *p*.001, η_p_^2^ < .29) and not in the very preterm group (*p*s > .05, η_p_^2^s < . 04).

#### Orienting-P2_diff_ (cue minus distractor)

**Figure 5.**
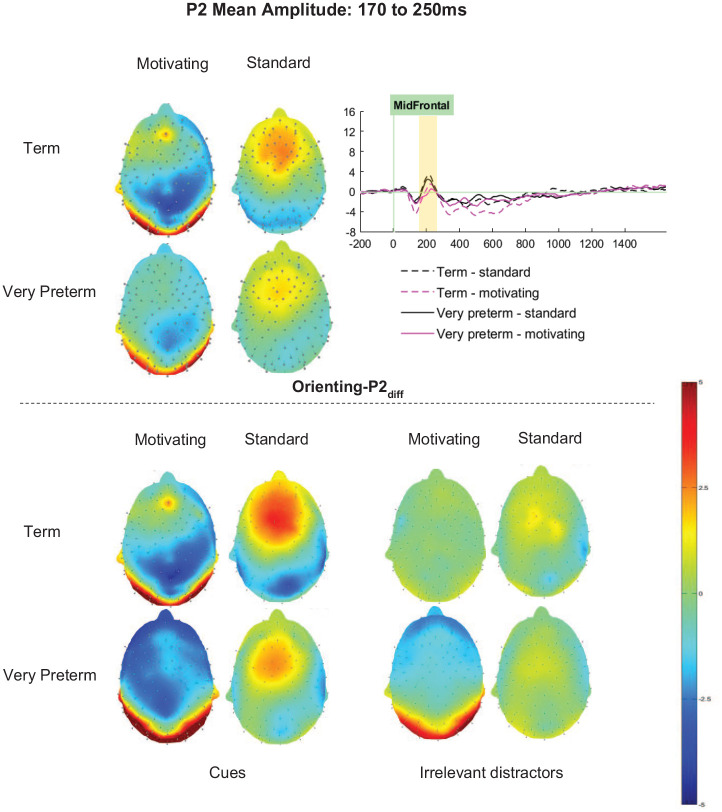
Orienting-P2 mean amplitude between 170 and 250ms.

For the Orienting-P2_diff_, significant main effects of Task (*F* [1, 66] = 57.76, *p*.001, η_p_^2^ = .67) and Cluster (*F* [2.83, 186.74] = 22.90, *p* < .001, η_p_^2^ = .26) were qualified by a significant Task by Cluster interaction (*F* [3.13, 206.57] = 3.87, *p* = .009, η_p_^2^ = .06). Similar topographical patterns were observed for each task, with Orienting-P2_diff_ amplitudes being maximal in the mid-frontal cluster. Orienting-P2_diff_ amplitudes were significantly higher for the standard variant of the task than the motivating variant at all clusters (*p*s < .01; see Figure 5). The interaction reflects that the effect of Task on Orienting-P2_diff_ was strongest in right and midline clusters (mid-frontal η_p_^2^ = .38; mid-central η_p_^2^ = .41; right-frontal η_p_^2^ = .39; right-central η_p_^2^ = .30), with smaller effects in the left-frontal and left-central clusters (η_p_^2^s < .20).

For Orienting-P2_diff_ there was no significant main effect of Group (*F* [1,66] = 1.09, *p* = .300, η_p_^2^ = .02). The interactions between Group and Task (*F* [1,66] = 0.31, *p* = .583, η_p_^2^ < .01), Cluster and Group (*F* [2.83, 186.74] = 2.39, *p* = .074, η_p_^2^ = .04) and Task, Cluster and Group (*F* [3.13, 206.57] = 1.13, *p* = .339, η_p_^2^ = .02) did not meet significance.

#### Orienting-P3a_diff_ (cue minus distractor)

For the Orienting-P3a_diff_ there was no significant main effect of Group (*F* [1,66] = 1.30, *p* = .258, η_p_^2^ = .02). However, there were significant main effects of Task (*F* [1,66] = 17.20, *p* < .001, η_p_^2^ = .21) and Cluster (*F* [2.87, 189.56] = 55.88, *p* < .001, η_p_^2^ = .46), and significant interactions between Group and Cluster (*F* [2.87, 189.56] = 3.38, *p* = .021, η_p_^2^ = .05), Task and Cluster (*F* [2.69, 177.50] = 25.79, *p* < .001, η_p_^2^ = .28), as well as a marginal interaction between Group and Task (*F* [1,66] = 3.83, *p* = .055, η_p_^2^ = .06). These were all qualified by a significant three-way interaction between Task, Group and Cluster (*F* [2.69, 177.50] = 3.65, *p* = .017, η_p_^2^ = .05).

**Figure 6.**
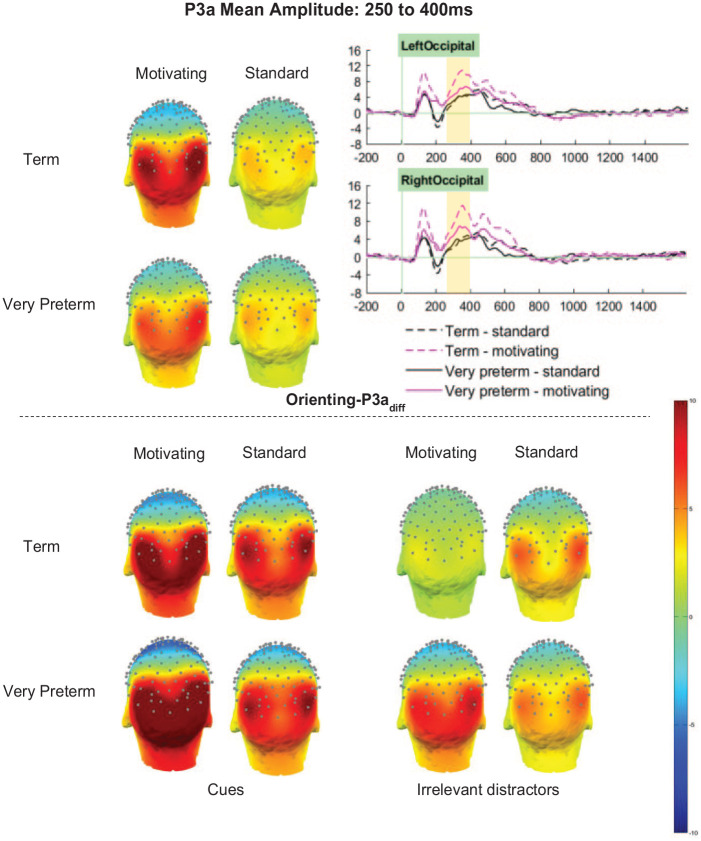
Orienting-P3a mean amplitude between 250 and 400 ms.

Orienting-P3a_diff_ amplitudes in the left-parietal and mid-parietal clusters were unaffected by Task or Group (*p*s > .1). In the right-parietal and mid-occipital clusters, amplitudes were significantly greater for the motivating task than the standard task (*p*s < .001), but did not differ between, or interact with, Group (*p*s > .1). In both the left and right occipital cluster, both main effects of task (LO *p* < .001, η_p_^2^ = .27; RO *p* < .001, η_p_^2^ = .31) and interactions between Task and Group (LO *p* = .016, η_p_^2^ = .09; RO *p* = .027, η_p_^2^ = .07) were observed (see Figure 6). Sidak-corrected pairwise comparisons showed that in both clusters, greater amplitudes in term-born relative to very preterm children were observed only in the motivating task (LO motivating *p* = .015, η_p_^2^ = .09; LO standard *p* = .701, η_p_^2^ < .01; RO motivating *p* = .025, η_p_^2^ = .07; RO standard *p* = .902, η_p_^2^ < .01). Meanwhile, in the left-occipital cluster between-task differences were only observed in the term-born children (term *p* < .001, η_p_^2^ = . 29; very preterm *p* = .089, η_p_^2^ = .04), but in the right-occipital cluster amplitudes were significantly greater for the motivating task in both groups (term *p* < .001, η_p_^2^ = .31; very preterm *p* = .031, η_p_^2^ = .07).

#### Orienting-P3b_diff_ (cue minus distractor)

**Figure 7.**
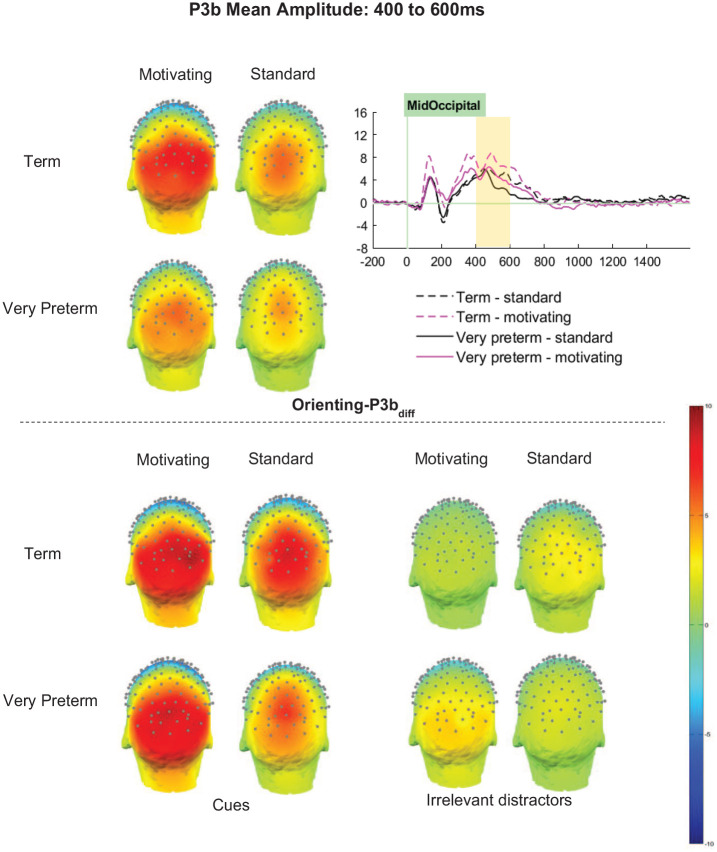
Orienting-P3b mean amplitude between 400 and 600ms.

Orienting-P3b_diff_ amplitudes were significantly greater for term-born children *(M* = 4.78 μV, *SD* = 0.37 μV) than those born very preterm (*M* = 3.26 μV, *SD* = 0.37 μV; *F* [1, 66] = 7.19, *p* = .009, η_p_^2^ = .10; see Figure 7). However, interactions between Group and Task (*F* [1,66] = 1.26, *p* = .259, η_p_^2^ = .02), Group and Cluster (*F* [2.98, 196.72] = 2.58, *p* = .055, η_p_^2^ = .04), and Group, Cluster and Task (*F* [3.01, 198.70] = 0.40, *p* = .757, η_p_^2^ < .01), did not meet significance.

Significant effects of Task (*F* [1,66] = 20.32, *p* < .001, η_p_^2^ = .24) and Cluster (*F* [2.98, 196.72] = 29.96, *p* < .001, η_p_^2^ = .31) were qualified by a significant interaction between Task and Cluster (*F* [3.01, 198.70] = 5.30, *p* = .002, η_p_^2^ = .07). Orienting-P3b_diff_ amplitudes were significantly greater for the motivating task than the standard task in all clusters, with effects strongest in clusters on the right (RP *p* < .001, η_p_^2^ = .30; RO *p* < .001, η_p_^2^ = .24) relative to those on the left (LP *p* = .003, η_p_^2^ = .13; LO *p* < .001, η_p_^2^ = .19) and at the midline (MP *p* = .020, η_p_^2^ = .08; MO *p* = .007, η_p_^2^ = .11).

## Discussion

### Key Findings

As hypothesized, across groups, children had higher hit rates and faster RT on “go” trials in the motivating task than the standard task, indicating better ability to sustain attention in the motivating task. Children born very preterm performed similarly to those born at term, which is not necessarily unexpected given the groups were well matched for parent-rated inattention (Retzler et al., 2019). Yet the attention-related neural activity associated with orienting in the tasks differed depending on preterm birth status, which may be indicative of disrupted processing of salience. This aligns to the findings from Jaeger et al. (2019), who found atypical oddball-P3 responses in low-risk preterm-born 5-to-6-year-olds, who performed equally well compared to their term-born peers on the task, and highlights the importance of using measures that are sensitive to unobservable processing differences.

### Implications

#### The Role of Motivation

Task performance was better in the motivating task variant and the dominant pattern across ERP components was that greater amplitudes, indicating more additional attentional resource, were observed in the motivating than standard task. Similarly, visual inspection of the RT distribution (see Supplemental Figure S3) indicated that in the motivating task relative to the standard task, fewer children had long RTs and there was an overall shift toward faster and less variable response times, suggesting the motivating features both reduced lapses in attention and improved arousal.

This performance enhancement occurred in both groups, indicating that it may be possible to apply arousal-regulation theories of ADHD (e.g., Cognitive-Energetic Model, Sergeant, 2005; Dynamic Developmental Theory, Savgolden et al., 2005) to very preterm groups and the study of attentional processing more generally in non-ADHD groups. It builds on evidence that incentives can improve attentional task performance in both ADHD and non-ADHD samples (Groom et al., 2010), showing that even without external reinforcers, increasing intrinsic motivation can have positive impacts on abilities to sustain attention in individuals without ADHD. Although this study wasn’t designed with the Self-Determination Theory (SDT) in mind, our findings provide supporting evidence that making a task more interesting, enjoyable, and satisfying, can stimulate better arousal regulation and attentional resource allocation to task-relevant stimuli, resulting in improved task performance. This has practical implications for the design and testing of interventions and strategies to support those with attention difficulties in educational settings and beyond. It remains unclear whether, as per the SDT, interventions that improve intrinsic motivation may be *more* effective at facilitating sustained attention than those that are incentive based, and further research is required to explore this further.

Between-task differences in ERPs were supportive of the interpretation that attention allocation was greater for the motivating task variant, where Orienting-P1_diff_, Orienting-P3a_diff_, Orienting-P3b_diff_ and Target-P1_diff_ amplitudes were greater, than the standard variant. The Orienting-P2_diff_ findings seem somewhat at odds with those observed in other components, with smaller amplitudes observed for the motivating than the standard task. P2 is a component considered to reflect perceptual matching between the stimulus presented and representations in memory. In the case of the posterior P2, which is usually elicited by figure detection paradigms, it is not unusual for amplitude to reduce as salience increases (Straube & Fahle, 2010). It has been speculated that this may occur because highly salient stimuli “pop-out” of a scene and are perceived effortlessly, thus perceptual matching requires less top-down control. It is plausible, therefore, that the reduced Orienting-P2_diff_ may also reflect a positive impact of manipulations to make the standard task more intrinsically motivating. Indeed, Orienting-P2_diff_ between-task differences were strongest in midline and right lateralized clusters, which may indicate additional resource was required to orient attention to the stimuli in the standard task that were not necessary in the motivating task.

#### Atypical Processing in Children Born Very Preterm

In spite of similar task performance in the term-born and very preterm children, for Orienting-P1_diff_ and Orienting-P3a_diff_, stronger between-task differences were observed in the term group than in the very preterm group, indicating that the term-born children were better able to increase the level of attentional resource when motivated than those born very preterm. Closer inspection of the topo plots for the absolute Orienting-P1 and Orienting-P3 elicited in response to cue trials and irrelevant distractors (see Figures 4 and 6), which were the parent components of the difference waves analyzed, may help us better understand this pattern of results. In the motivating task, while term-born children did not show much activity to irrelevant distractors, those born very preterm did; thus in the very preterm children the difference between the activity evoked by cue stimuli and that evoked by irrelevant distractors was smaller. This indicates that very preterm children may allocate a similar absolute level of resource to processing the cue stimuli in the motivating task, with the smaller additional resource for cue relative to distractor stimuli being driven by less suppression of activity to task-irrelevant stimuli when motivation is high. This supports other emerging findings that suggest atypical processing in very preterm children may only be observed under certain conditions (Retzler et al., 2022), and may explain why such differences in neural processing do not negatively impact task performance.

This interpretation aligns with findings that young adults born preterm/very low birth weight showed increased P3 amplitudes to non-targets, which researchers have interpreted as difficulties with selective attention (Aasen et al., 2016). What is interesting, is that the group-related interactions we observed were not ubiquitous across all components, and in the standard task variant, absolute amplitudes of components evoked by irrelevant distractors were very similar in both groups. Perhaps then, it is only when salience is high that those born very preterm allocate significant resource to processing all stimuli, whether task-relevant or not.

Other findings have indicated disruptions to the salience network in preterm-born samples. At rest, significantly decreased right salience network functional connectivity has been observed in 6-year-olds born very preterm (Cho et al., 2022), and adults born at very preterm gestations displayed less connectivity between the salience and default mode networks (White et al., 2014). Similarly, in an extremely preterm sample of 10-year-olds, disruption was observed to the salience, default mode and dorsal attention networks (Padilla et al., 2023). Given the salience network is considered to be responsible for switching between central executive network (CEN) and default mode network (DMN) activation (Uddin, 2015), it may be that poor connectivity results in less effective activation and deactivation of the neural networks that support efficient task processing. Of interest, Liddle et al. (2011) found that in children with ADHD, DMN *de*activation was only effective when motivational salience is high, and proponents of the SDT postulate that intrinsic motivation recruits the salience and central executive networks, while suppressing the DMN (Di Domenico & Ryan, 2017). Taken together, we suggest that in conditions with high motivational salience, and in populations where salience network processing is atypical, such as in those born very preterm, the salience network may be less effective at switching between networks in response to task demands, and thus the DMN deactivation may extend to both task-relevant and task-irrelevant stimuli. As such, when motivated, the resource available for task-relevant processing allows improved task performance, but limited suppression occurs for task-irrelevant processing. It is unclear what behavioural impacts this may have, and whether the cognitive demands of elevated processing of task-irrelevant stimuli may result in greater fatigue, but this is an interesting avenue for future research.

### Strengths and Limitations

As the first study to investigate the impact of adaptations designed to stimulate intrinsic motivation on a behavioral and neural level in term-born and very preterm children in middle childhood, these findings extend our understanding of atypical development following very preterm birth and of approaches that could support those who struggle to sustain attention more broadly. Use of EEG allowed identification of differences unobservable using task-performance measures.

The PATCH Study benefits from a term-born comparison group with a similar range of parent-rated inattention to the very preterm group, who were representative of the population from which they were drawn in terms of birth weight, gestational age and sex. Unfortunately, due to a combination of time constraints, technical issues and intolerance to the EEG procedure, not all children recruited to the PATCH study had EEG and behavioral data available for the two variants of the task (see Supplementary Material for full comparison). Compared with those without available data, the ERP analysis sub-sample were of higher IQ and higher gestational age, but well-matched on sex, age, ethnicity, socio-economic status, and, importantly, parent-rated inattention. Moreover, the same group differences between term-born and very preterm children were observed in the sub-sample and full sample (only in age and IQ) and the resulting sub-sample provided equally sized groups that were well powered for investigation of differences between groups and tasks. Larger samples would offer more power to detect 2- and 3- way interactions, and the absence of any group-by-task interactions (in the behavioral data in particular) should be treated cautiously, although any such patterns are likely to be so small (frequently η_p_^2^ < .01 in our data) as to be of limited practical or clinical importance. Similarly, while the patterns of exploratory correlational analyses (see Supplemental Tables S1 and S2 in Supplementary Materials) largely align with the interpretation that greater amplitudes reflected better allocation of attention, resulting, in turn, in better performance, a larger sample would have provided the power needed to fully understand the relationships between orienting activity and subsequent target processing and performance.

Certain aspects of our study design limit the conclusions that can be drawn. For example, the visual discrepancy between the stimuli presented in the two task variants creates some uncertainty in our interpretation. To better allow comparison of attention-related activity between tasks and reduce the impact of between-task differences in background luminance, and stimulus color and complexity, difference waves were computed (Kappenman et al., 2021). However, it should be noted that in the case of the Orienting_diff_ wave, the difference wave approach does not fully isolate the neural activity associated with attention allocation in each task. While subtraction of the ERP elicited by the cue stimuli from the averaged ERP of the 10 distractor stimuli, should reduce the contribution of visual properties that are *common* within a task, stimulus-specific differences between the cue ERP and averaged distractor ERP may introduce some noise into the difference waveform. It therefore remains possible that the differences in visual salience contribute to the task-related effects and interactions. Even so, both semantic and physical properties of stimuli are likely to impact processing; for example, Bachman et al. (2020) found that increasing the physical salience of stimuli can promote *faster* orienting, while increasing value-driven salience through offering feedback and speed and accuracy -contingent rewards can lead to *strengthened* attentional orienting. Thus, while we assume our findings are driven by more than visual properties alone, or, similarly, more than feedback alone, within this study it is not possible to determine the extent to which individual task manipulations contributed to the pattern of results. Studies that systematically vary individual components of intrinsic motivation, and control for visual salience, are required to better understand their independent contributions.

In line with the wider literature, our interpretations assume the ERP and task performance metrics are sensitive to changes in arousal (e.g. James et al., 2016), however, this interpretation could have been strengthened by including a physiological measure of autonomic responses such as skin conductance level. Moreover, while anecdotally, researchers reported that children did express a preference for the motivating task variant, no measures of subjective enjoyment were recorded, preventing any confirmatory analysis of our manipulations. Theoretically speaking, further research is required to assess the extent to which subjectivity in individual preferences may impact intrinsic motivation. Studies that use methods that aim to distinguish between the impact of objective elements of manipulation (i.e. presence vs. absence of a sound) and subjective preference (i.e. the extent to which the feedback is motivating for the individual) would be required to investigate this further.

## Conclusion

Taken together, these findings show that manipulations that aim to increase intrinsic motivation can promote sustained attention in term-born and very preterm children, and thus strategies that aim to support learning and concentration in educational and other settings with lengthy tasks, could benefit from designing them to be enjoyable, satisfying and to reinforce competence. Under conditions of high motivation, however, children born very preterm may be less likely than those born at term to use top-down control to selectively orient and attend to only task-relevant stimuli. Future research is required to fully understand the conditions under which very preterm born children may struggle to adjust their attentional focus, and whether there are any adverse impacts on tiredness or subsequent performance.

## Supplemental Material

sj-pdf-1-jad-10.1177_10870547251313888 – Supplemental material for The Impact of Motivation on Sustained Attention in Very Preterm and Term-born Children: An ERP Study Supplemental material, sj-pdf-1-jad-10.1177_10870547251313888 for The Impact of Motivation on Sustained Attention in Very Preterm and Term-born Children: An ERP Study by Jenny Retzler, Madeleine J. Groom, Samantha Johnson and Lucy Cragg in Journal of Attention Disorders
