# Supplementary material for: The Impact of Motivation on Sustained Attention in Very Preterm and Term-born Children: An ERP Study
Source: J Atten Disord. 2025 Jan 28;29(7):569–88. doi: 10.1177/10870547251313888 (PMC11956374; doi:10.1177/10870547251313888)
Supplement: sj-pdf-1-jad-10.1177_10870547251313888 – Supplemental material for The Impact of Motivation on Sustained Attention in Very Preterm and Term-born Children: An ERP Study [file sj-pdf-1-jad-10.1177_10870547251313888.pdf]

Supplementary methodological information

Sample details

Full details of recruitment procedures are described in Retzler et al. (2019), but Figure S1 provides a summary of the participant flow from initial recruitment to the inclusion of participants in the event-related potential (ERP) analysis sub-sample. The full study sample comprised 65 children born very preterm (VP) and 48 children born at term. Of these 31 VP and 14 term-born children did not complete both of the CPT-AX tasks on which this analysis is performed (due to time constraints (9 VP and 3 term), technical issues (4 VP) or intolerance to the EEG fitting procedure (9 VP and 3 term), completion of only one CPT-AX task (9 VP and 8 term)). The resulting final sub-sample for this analysis comprised 34 VP and 34 term-born children.

Figure S1: Participant flow from recruitment to ERP analysis

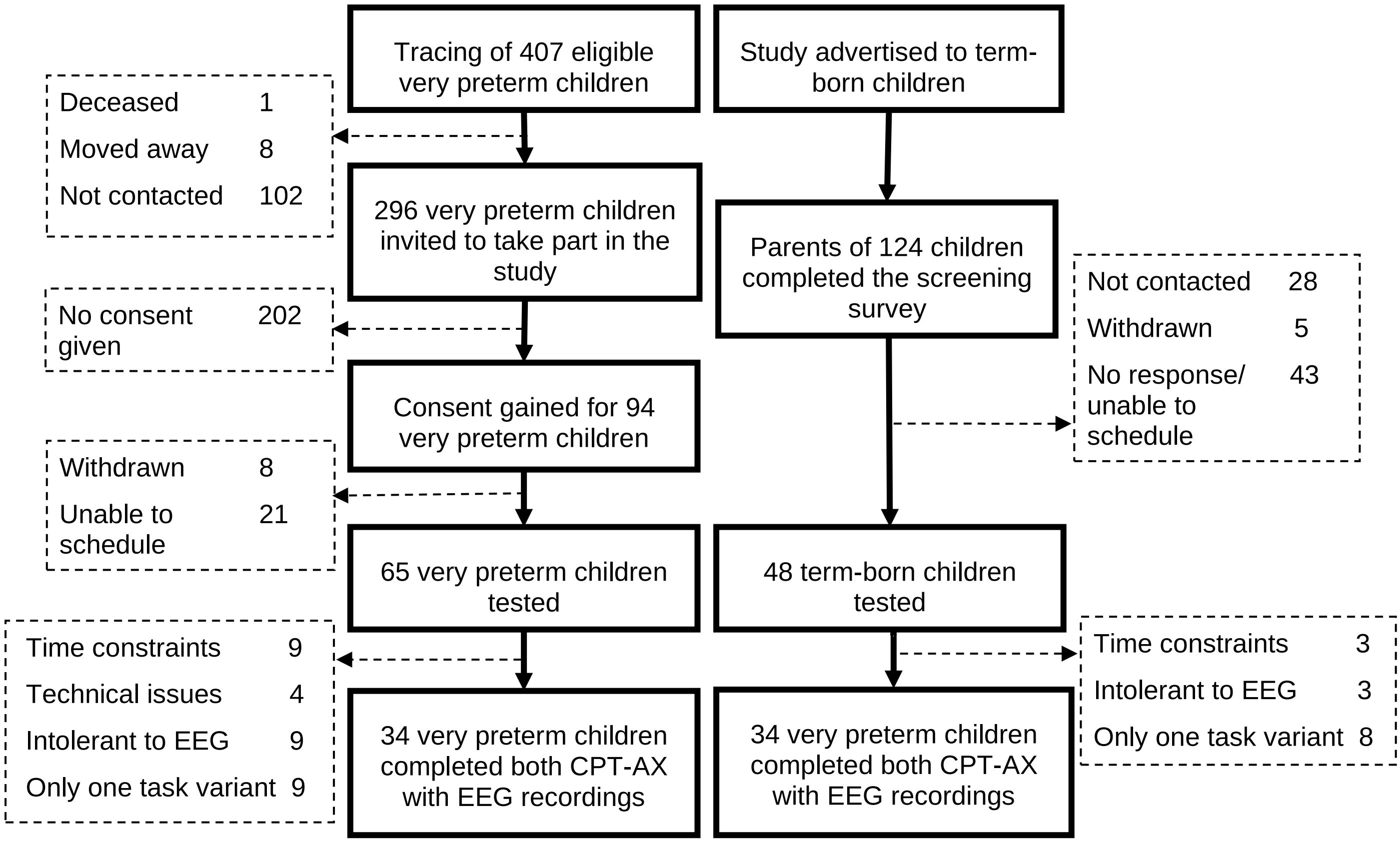

Table S1 reports sample characteristics for children in the full sample and those included in the ERP analysis sub-sample, and the differences between term-born and VP children in each. Differences between children from the full sample who were included vs. not included in the ERP analysis were assessed using *t*-tests for continuous variables, and chi-squared analysis for categorical variables. Children included in the current ERP analysis did not differ from those without available data on age, sex, ethnicity, socio-economic status, or, importantly, Conner's 3 and SWAN scores of inattention and hyperactivity (p>0.1 in all cases). However, compared to those who did not have data available for the ERP analysis, those who did were born at significantly later gestational age (included M = 35^{+0} weeks, SD =5^{+1} weeks; data unavailable M = 33^{+0} weeks, SD = 5^{+1} weeks; p=.044), and had significantly higher IQ scores (included M = 108.00 points, SD = 11.80 points; data unavailable M = 101.72 points, SD = 14.33 points, p=.015).

The same group differences between term-born and VP children were observed in both the full sample and the sub-sample included in the current ERP analysis. Compared with term-born children VP children were significantly older and had significantly lower IQ but were well-matched on other variables (see Table S1).

Table S1: Sample characteristics for children included in the ERP analysis

	**Full sample**			**ERP Analysis sub-sample**		
	Very Preterm (n=65[a])	Term (n=48[a])	p	Very Preterm (n=34[a])	Term (n=34[a])	p
Participant characteristics						
Gestation (weeks)						
Mean (*SD*)	29^{+6} (1^{+6})	40^{+0} (1^{+1})	-	30^{+1} (1^{+6})	40^{+0} (1^{+1})	-
Range	26 to 32	37 to 42		26 to 32	37 to 42	
Age (years)						
Mean (*SD*)	10.1 (0.9)	9.6 (1.0)	.006*	10.04 (0.92)	9.54 (1.04)	.040*
Range	8.0 to 11.0	8.0 to 11.7		8.41 to 11.41	8.0 to 11.5	
FSIQ-2[b]						
Mean (*SD*)	101.1 (13.9)	111.1 (9.9)	<.001*	103.5 (12.74)	112.6 (8.7)	<.001*
Range	67 to 131	83 to 127		78 to 131	89 to 127	
Score <70 *n*(%)	1 (1.5%)	0		0	0	
Demographics, *n*(%)						
Sex						
Male	36 (55.4%)	26 (54.2%)	.898 *n.s.*	18 (52.9%)	18 (52.9%)	>.999 *n.s.*
Female	29 (44.6%)	22 (45.8%)		16 (47.1%)	16 (47.1%)	
Ethnicity [c]						
White	47 (82.3%)	42 (87.5%)	.855 *n.s.*	27 (87.1%)	28 (84.8%)	.620 *n.s.*
Mixed	7 (12.3%)	4 (8.3%)		4 (12.9%)	4 (12.1%)	
Asian	1 (1.8%)	1 (2.1%)		0	0	
Black	1 (1.8%)	1 (2.1%)		0	1 (3.0%)	
Chinese	0	0		0	0	
Other	1 (1.8%)	0		0	0	
Socio-economic Status (SES)						
Low SES	12 (18.5%)	13 (27.1%)	.074 *n.s.*	7 (20.6%)	9 (26.5%)	.556 *n.s.*
Middle SES	25 (38.5%)	9 (18.8%)		12 (35.3%)	8 (23.5%)	
High SES	28 (43.1%)	26 (54.2%)		15 (44.1%)	17 (50.0%)	
Conner's 3 ADHD symptom scores						
Conner's 3 *T*-scores, mean *(SD)*						
DSM ADHD/I	62.11 (15.48)	57.79 (13.51)	.136 *n.s.*	59.62 (14.40)	56.88 (11.95)	.397 *n.s.*

DSM ADHD/C	61.63 (14.42)	58.48 (14.08)	.399 *n.s.*	60.82 (15.72)	56.97 (13.84)	.287 *n.s.*
Inattention	60.71 (15.64)	57.13 (12.29)	.215 *n.s.*	57.97 (14.35)	56.18 (13.35)	.595 *n.s.*
Hyperactivity/ Impulsivity	62.15 (16.24)	59.06 (14.47)	.297 *n.s.*	61.03 (16.03)	58.24 (14.37)	.452 *n.s.*
IA-HI correlation, *r*	.78	.83	.233 *n.s.*	.81	.83	.810 *n.s.*
Conner's 3 scores above clinical cut offs, *n*(%)						
DSM ADHD/I	22 (34.4%)	12 (25.0%)	.286 *n.s.*	9 (26.5%)	7 (20.6%)	.567 *n.s.*
DSM ADHD/C	21 (32.3%)	13 (27.1%)	.549 *n.s.*	9 (26.5%)	8 (23.5%)	.779 *n.s.*
SWAN symptom scores [d]						
Inattention						
Mean (*SD*)	-.068 (10.89)	-4.67 (12.22)	.080 *n.s.*	-2.65 (9.87)	-5.39 (11.74)	.316 *n.s.*
Range	-26 to 26	-27 to 20		-26 to 21	-27 to 18	
Hyperactivity/ Impulsivity						
Mean (*SD*)	-2.86 (11.13)	-6.71 (12.55)	.099 *n.s.*	-3.84 (10.32)	-7.39 (12.70)	.226 *n.s.*
Range	-27 to 25	-27 to 27		-27 to 14	-27 to 27	

Note: Age reflects chronological age for VP children. Continuous variables were compared using independent samples t-tests, rank variables were compared using Pearson's chi-square, correlations were compared using Fischer's r-to-z. SD=standard deviation, FSIQ-2= two-subtest full scale intelligence quotient calculated using the Wechsler Abbreviated Scale for Intelligence. IA-HI correlation = correlation between inattentive (IA) and hyperactive-impulsive (HI) symptoms as measured using the Conner's 3 subscale *T*-scores. *p<0.05, *n.s.*= not significant. [a] accurate unless otherwise indicated. [b] Full sample: VP = 65, Term = 47. ERP sub-sample: VP = 34, Term = 33. [c] Full sample: VP = 57, Term = 48. ERP sub-sample: VP = 31, Term = 33. [d] Full sample: VP = 57, Term = 48. ERP sub-sample: VP = 31, Term = 34.

Task details

Figure S2 shows the stimuli that were used in the standard and motivating tasks.

Figure S2: Task stimuli

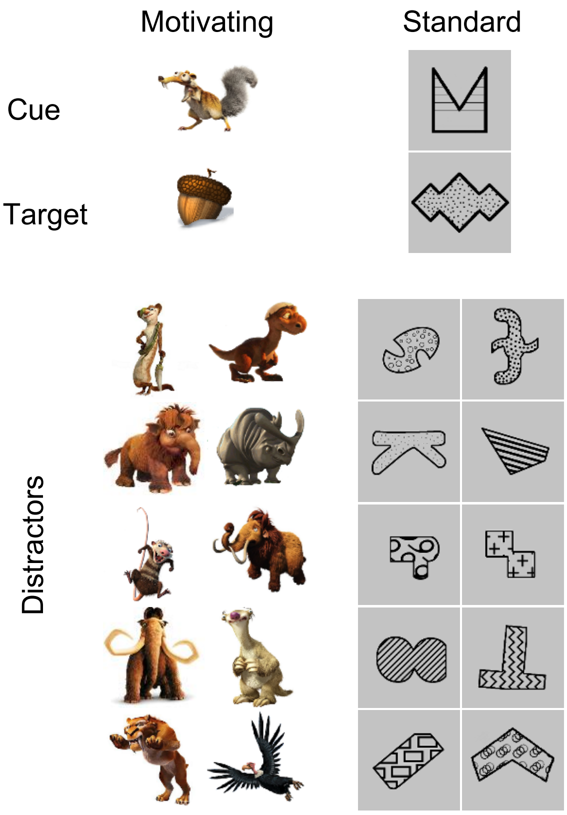

ERP preprocessing

Averaging across clusters

Electrode positions were based upon the 10/5 system, an extension of the traditional 10/20 system (Oostenveld and Praamstra, 2001), with data collected at 117 sites (Fp1, Fpz, Fp2, AFp3, AFp4, AF7, AF3, AF1, AFz, AF2, AF4, AF8, AFF5h, AFF3, AFF1h, AFF2h, AFF4, AFF6h, F7, F5, F3, F1, Fz, F2, F4, F6, F8, FFT7h, FFC5h, FFC1h, FFC2h, FFC4h, FFC6h, FFT8h, FT7, FC5, FC3, FC1, FCz, FC2, FC4, FT8, FTT7h, FCC5h, FCC3h, FCC1h, FCC2h, FCC4h, FCC6h, FTT8h, T7, C5, C3, C1, Cz, C2, C4, C6, T8, TTP7h, CCP5h, CCP3h, CCP1h, CCP2h, CCP4h, CCP6h, TTP8h, TP7, CP5, CP3, CP1, CPz, CP2, CP4, CP6, TP8, TPP7h, CPP5h, CPP3h, CPP1h, CPP2h, CPP4h, CPP6h, TPP8h, P7, P5, P3, P1, Pz, P2, P4, P6, P8, PPO5h, PPO3, PPO1h, PPO2h, PPO4, PPO6h, PO7, PO3, PO1, POz, PO2, PO4, PO8, POO1, POO2, POO4, O1, Oz, O2).The waveforms were averaged across electrodes into clusters as follows: Left frontal = F3, FFC5H, FFC3H, AFF5h, AFF3; Mid frontal = Fz, FFC1h, FFC2h, AFF1h, AFF2h; Right frontal = F4, FFC4h, FFC6h, AFF4,

AFF6h; Left central = C3, CCP5h, CCP3h, FCC5h, FCC3h; Mid central = Cz, CCP1h, CCP2h, FCC1h, FCC2h; Right central = C4, CCP4h, CCP6h, FCC4h, FCC6h; Left parietal = P3, PPO5h, PPO3, CPP3h, CPP5h; Mid parietal = Pz, PPO1h, PPO2h, CPP1h, CPP2h; Right parietal = P4, PPO4, PPO6h, CPP4h, CPP6h; Left occipital = O1, PO1, PO3, POO3; Mid occipital = Oz, POz, POO2, POO1; Right occipital = O2, PO2, PO4, POO4.

Artefact rejection procedure

Artefact rejection, conducted using FASTER (Nolan et al., 2010), defined contaminated data as any with a z score of ±3 for that metric. First, deviant channels are identified based on; (i) low mean correlations with neighbouring channels, (ii) high channel variance, and (iii) atypical Hurst exponent values. These channels are then interpolated, removing the effect of any bad channels. In step two, deviant epochs are identified based on; (i) high amplitude ranges within epochs, (ii) extreme deviation from the mean channel average, and (iii) high variance. These epochs are then removed from the data, removing the effect of epochs contaminated by artefacts such as movement. In step three, independent components analysis is conducted using the Infomax algorithm and deviant components are identified by; (i) strong correlations with EOG electrodes, (ii) activity observed in only a single electrode, (iii) activity with a flat power spectrum (white noise), (iv) atypical Hurst exponent values, and (v) the median gradient value of the IC timecourse. These are then subtracted from the data, removing the effect of artefacts such as eye blinks, and high amplitude single-electrode pop-off. Finally, in step four, deviant recordings from specific channels within specific epochs are identified based on; (i) high variance of specific channels within each epoch, (ii) the median gradient to detect high frequency activity, (iii) high amplitude ranges of the channel, (iv) deviation of that channel from the channel average within the epoch. Bad channels within epochs were then interpolated to remove the effects of transient artefacts within epochs.

Supplementary results information

Response time distribution

Visual inspection of the distribution of median response times in the standard and motivating tasks suggests that between-task differences in RT were driven by a range of factors. Figure S3 shows that more children had long RTs in the standard vs. the motivating task, suggesting fewer children experienced lapses in attention during the motivating task and that they were more engaged in the task. There was also an overall shift in the distribution of response times to faster and less variable median RTs in the motivating task relative to the standard task, suggesting overall higher arousal in the task.

Figure S3: Histograms of the median response time distribution in each task

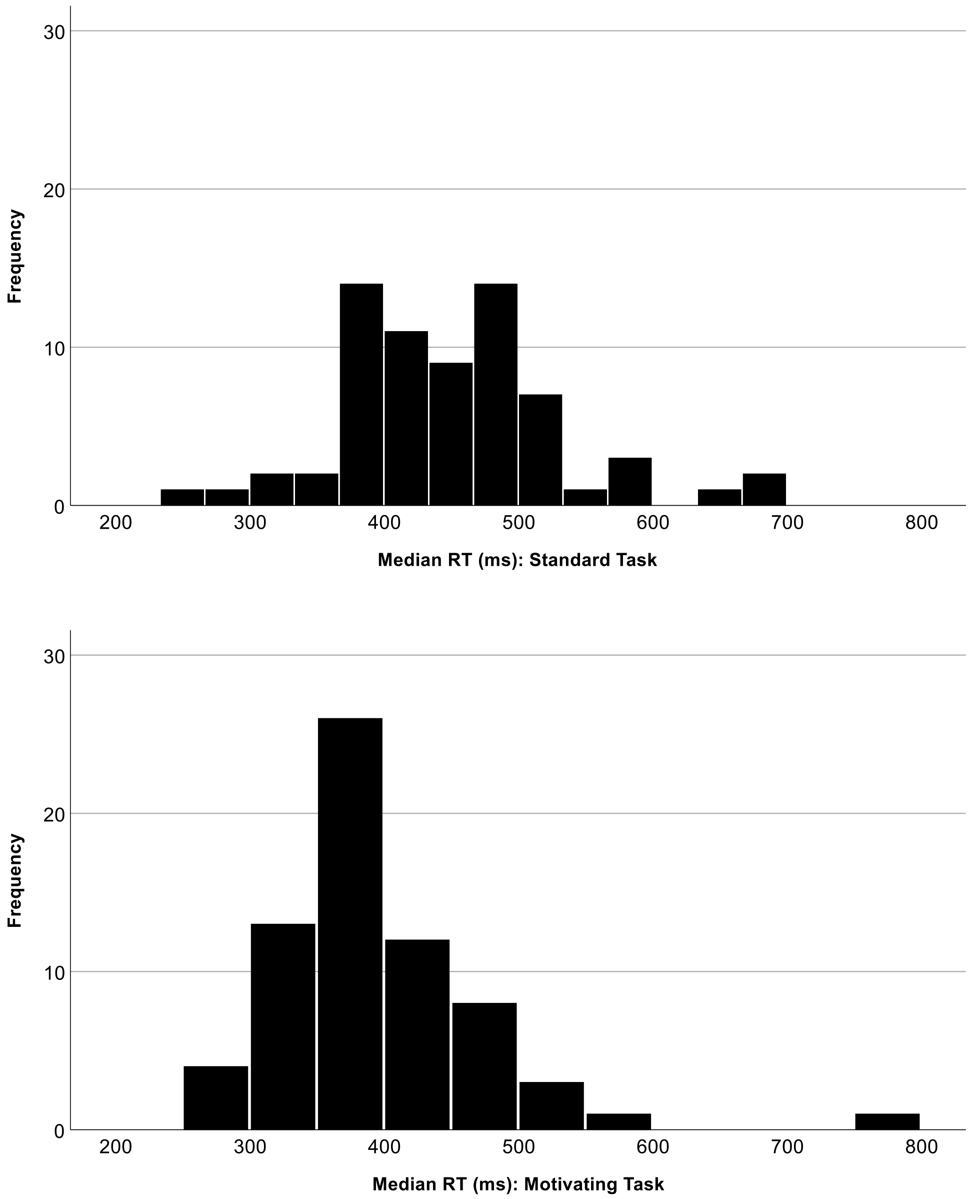

Age-related effects in ERPs

For all ERP analyses, effects of age and interactions between age and other variables did not meet the threshold for significance (*p*s>.01) and thus all effects are reported from the ANOVA without the covariate. These null effects and interactions are reported in full below.

Age had no significant main effect for Orienting-$P1_{diff}$ ($F(1,65) = 3.65$, p=.060, η_p^2=.05), Orienting-$P2_{diff}$ ($F(1,65) = 0.01$, p=.912, η_p^2<.01), Orienting-$P3a_{diff}$ ($F(1,65) = 0.02$, p=.880, η_p^2<.01), Orienting-$P3b_{diff}$ ($F(1,65) = 1.33$, p=.253, η_p^2=.02), Target-$P1_{diff}$ ($F(1, 65) = 0.32$, p=.571, η_p^2< .01), Target-$P2_{diff}$ ($F(1, 65) = 0.61$, p=.437, η_p^2< .01), Target-$P3a_{diff}$ ($F(1, 65) = 0.38$, p=.543, η_p^2< .01), or Target-$P3b_{diff}$ ($F(1, 65) = 1.57$, p=.215, η_p^2< .01).

Age did not interact significantly with task for Orienting-P1$_{diff}$ (F(1,65) = 3.13, p=.081, η_p^2=.05), Orienting-P2$_{diff}$ (F(1,65) =3.29, p=.075, η_p^2=.05), Orienting-P3a$_{diff}$ (F(1,65) = 4.90, p=.030, η_p^2=.07), Orienting-P3b$_{diff}$ (F(1, 65) = 4.97, p=.048, η_p^2= .06), Target-P1$_{diff}$ (F(1, 65) = 0.06, p=.806, η_p^2< .01), Target-P2$_{diff}$ (F(1, 65) = 0.59, p=.446, η_p^2< .01), Target-P3a$_{diff}$ (F(1, 65) = 0.12, p=.736, η_p^2< .01), or Target-P3b$_{diff}$ (F(1, 65) = 0.39, p=.533, η_p^2< .01).

Age did not interact significantly with location for Orienting-P1$_{diff}$ (F(2.93, 190.65) = 2.99, p = 0.033, η_p^2= .04), Orienting-P2$_{diff}$ (F(2.81, 182.77) = 0.30, p=.810, η_p^2<.01), Orienting-P3a$_{diff}$ (F(2.84, 184.62) = 0.70, p=.543, η_p^2=.01), Orienting-P3b$_{diff}$ (F(2.96, 192.08) = 0.44, p=.723, η_p^2<.01), Target-P1$_{diff}$ (F(2.61, 169.79) = 0.29, p=.802, η_p^2< .01), Target-P2$_{diff}$ (F(2.66, 172.78) = 0.14, p=.917, η_p^2< .01), Target-P3a$_{diff}$ (F(2.59, 168.22) = 0.59, p=.600, η_p^2< .01), or Target-P3b$_{diff}$ (F(2.55, 165.65) = 2.55, p=.067, η_p^2< .04).

There was no significant three-way interaction with age, task and location for Orienting-P1$_{diff}$ (F(2.56, 168.94) = 3.24, p = 0.030, η_p^2= .05), Orienting-P2$_{diff}$ (F(3.12, 202.68) = 0.86, p=.51, η_p^2=.01), Orienting-P3a$_{diff}$ (F(2.72, 176.66) = 2.29, p=.087, η_p^2=.03), Orienting-P3b$_{diff}$ (F(3.05, 198.25) = 1.99, p=.115, η_p^2=.03, Target-P1$_{diff}$ (F(2.82, 183.38) = 0.57, p=.628, η_p^2< .01), Target-P2$_{diff}$ (F(3.17, 206.22) = 0.38, p=.781, η_p^2< .01), Target-P3a$_{diff}$ (F(3.43, 223.18) = 1.19, p=.316, η_p^2= .02), or Target-P3b$_{diff}$ (F(3.39, 220.09) = 0.96, p=.421, η_p^2= .02).

Target-evoked components

Target-P1$_{diff}$ (cued target minus uncued target)

Target-P1$_{diff}$ amplitudes were significantly greater for the motivating task variant (M = 2.01 μV, SD = 0.24 μV) than the standard task (M = 0.63 μV, SD = 0.27 μV; F(1,66) = 21.20, p<.001, η_p^2=.24). However, there were no significant main effects of Group (F(1, 66) = 0.21, p=.651, η_p^2< .01) or Cluster (F(2.63, 173.56) = 2.22, p=.096, η_p^2= .03). And no interactions met the threshold for significance (Task by Group F(1, 66) = 0.25, p=.618, η_p^2< .01; Task by Cluster F(2.85, 188.33) = 2.41, p=.072, η_p^2= .04; Group by Cluster F(2.63, 173.56) = 0.54, p=.632, η_p^2< .01; Task by Group by Cluster F(2.85, 188.33) = 0.85, p=.466, η_p^2= .01).

Target-P2$_{diff}$ (cued target minus uncued target)

There were no significant main effects of Task (F(1, 66) = 3.02, p=.087, η_p^2= .04) or group (F(1, 66) = 0.50, p=.484, η_p^2<.01) for the Target-P2$_{diff}$. There was a significant main effect of Cluster (F(2.66, 175.97) = 28.49, p<.001, η_p^2= .30) which was qualified by a significant interaction between Task and Cluster (F(3.19, 210.30) = 5.38, p=.001, η_p^2= .08). Target-P2$_{diff}$ amplitudes were similar for both tasks in the left frontal, central frontal and mid central clusters (ps>.05). In the left central cluster, amplitudes were positive and significantly greater for the motivating task than the standard task (p=.039, η_p^2= .06). In the right frontal cluster,

however, the difference wave was negative, reflecting that activity during stimulus categorization in this part of the scalp was greater for the no-go presentation of the X stimulus than the go presentation. The magnitude of this was greater for the motivating task than the standard task (*p*=.002, η_p^2= .14). In the right central cluster, mean amplitudes were small and positive for the standard task, but negative and of greater magnitude for the motivating task (*p*=.008, η_p^2= .10).

Interactions between Cluster and Group (F(2.66, 175.97) = 0.36, *p*=.759, η_p^2<.01) and Task, Cluster and Group (F(3.19, 210.30) = 1.57, *p*=195, η_p^2= .02) were not significant.

Target-P3a$_{diff}$ (cued target minus uncued target)

For Target-P3a$_{diff}$ there was no significant main effect of Group (F(1, 66) = 0.23, *p*=.634, η_p^2<.01), or task (F(1,66) = 3.25, *p*=.076, η_p^2=.05) and no interaction between Task and Group (F(1, 66) = 0.32, *p*=.574, η_p^2< .01). Target-P3a$_{diff}$ amplitude varied significantly by Cluster (F(2.59, 170.82) = 25.01, *p*<.001, η_p^2=.28) and this effect of location was qualified by significant interactions with both Task (F(3.42, 225.63) = 2.79, *p*=.035, η_p^2=.04) and Group (F(2.59, 170.82) = 3.17, *p*=.032, η_p^2=.05).

Target-P3a$_{diff}$ was significantly higher for the motivating task than the standard task only in the right (*p*=.047, η_p^2=.06) and left parietal clusters (*p*<.001, η_p^2=.22). Although there were no significant between-group differences at any location (*p*s>.05), the effect of location was slightly stronger in very preterm (*p*<.001, η_p^2=.48) relative to term-born children (*p*<.001, η_p^2=.40).

For both groups, amplitudes at occipital clusters did not differ from one another, nor did those in bilateral parietal clusters (*p*s>.05). However, in those born at term, more right lateralization was observed, whereby amplitudes in the right and midline parietal clusters were significantly greater than those in the occipital clusters and left-parietal cluster (*p*s<.05), which were all similar in magnitude (*p*s>.05).

In those born very preterm, the distribution was more mid-parietal, with amplitudes in this cluster being significantly stronger than in all other locations (*p*s<.001), including the right parietal cluster. The three occipital clusters were of significantly smaller magnitude than the parietal clusters (*p*s<.05) with the exception of the right parietal and left occipital cluster (*p*>.05).

There was no significant three-way interaction between Group, Task and Cluster (F(3.42, 225.63) = 1.11, *p*=.351, η_p^2=.02).

Target-P3b$_{diff}$ (cued target minus uncued target)

For Target-P3b$_{diff}$ there were no significant main effects of Group (F(1, 66) = 0.83, p=.366, η_p^2=.01), or Task (F(1,66) = 3.31, p=.073, η_p^2=.05) and no significant interaction between the two (F(1, 66) = 0.18, p=.676, η_p^2< .01). Target-P3b$_{diff}$ amplitudes differed significantly by Cluster (F(2.50, 164.91) = 79.90, p<.001, η_p^2=.55). While Cluster did not interact with Group (F(2.50, 164.91) = 0.74, p=.507, η_p^2=.01), the main effect of Cluster was, however, qualified by a significant interaction between Task and Cluster (F(3.43, 226.40) = 4.84, p=.002, η_p^2=.07). Across tasks and for both tasks individually, Target-P3b$_{diff}$ amplitudes measured at parietal locations were small, but positive, while those measured at occipital locations were negative and of greater magnitude. Those measured at parietal locations did not differ significantly between tasks (ps>.05) while those measured at occipital locations were of significantly greater magnitude in the motivating task variant (for all occipital locations ps<.05, η_p^2s=.08).

Relationships between ERPs and task performance

In those born very preterm, better performance on the standard task variant was associated with larger amplitudes of the later P3a and P3b components. Specifically, larger Orienting-P3a$_{diff}$ related to less variability in response times (r = -.46, p = .006), while larger Orienting-P3b$_{diff}$ related to faster response times (r = -.35, p = .040), and larger Target-P3a$_{diff}$ related to fewer commission errors (r = -.35, p = .042) and marginally quicker response times (r = -.34, p = .052). In the motivating task variant, although Orienting-P3a$_{diff}$ and Orienting-P3b$_{diff}$ were marginally related to higher hit rates (Orienting-P3a$_{diff}$ r = .34, p = .050; Orienting-P3b$_{diff}$ r = .32, p = .062), performance was also associated with an earlier component of target processing, with greater amplitudes of Target-P2$_{diff}$ relating to significantly quicker (r = -.39, p = .022) and less variable (r = -.39, p = .023) responding.

In those born at term, on the standard task variant we observed moderate-to-strong associations between better task performance and larger amplitudes of the Orienting-P3b$_{diff}$ (hit rate r = .46, p = .007; response time variability r = -.53, p = .001; commission errors r = -.41, p = .016). Faster response time on this task showed trends towards a similar association with greater amplitudes of the late component of Orienting-P3a$_{diff}$ (r = -.31, p = .076). However, faster RT was also marginally associated with *smaller* amplitudes of the earlier component of Target-P1$_{diff}$ (r = .34, p = .053) and the later Target-P3b$_{diff}$ (r = .30, p = .086). In the motivating task, the strongest correlations between task performance and neural processing were in the early processing of the cue component, whereby fewer commission errors were made in the term-born children who had *smaller amplitudes* of Orienting-P1$_{diff}$ (r = .40, p = .019) and larger amplitudes of the Orienting-P2 (r = -.36, p =

.037), in addition to marginally larger Target-P3a$_{diff}$ amplitudes (*r* = -.32, *p* = .063). Less response time variability in the motivating task was also marginally related to greater P3b$_{diff}$ amplitudes (Orienting-P3b$_{diff}$ *r* = -.32, *p* = .065; Target-P3b$_{diff}$ (*r* = -.32, *p* = .079).

These findings largely align with the interpretation that greater amplitudes reflect better allocation of attention, resulting in better performance, as well as that the P3 task components are more closely linked to task performance.

Table S2: Correlations between task performance and ERPs on the standard task variant

		Term											
		Hits	RT	RTV	Comm. errors	Orienting-P1$_{diff}$ [a]	Orienting-P2$_{diff}$ [b]	Orienting-P3a$_{diff}$ [c]	Orienting-P3b$_{diff}$ [d]	Target-P1$_{diff}$ [e]	Target-P2$_{diff}$ [f]	Target-P3a$_{diff}$ [g]	Target-P3b$_{diff}$ [h]
Very Preterm	Hits		-.12<br>.598	**-.36<br>.034***	**-.79<br><.001***	-.16<br>.377	-.18<br>.297	.076<br>.668	**.46<br>.007**	-.14<br>.404	-.03<br>.884	.08<br>.669	.16<br>.381
	RT	-.11<br>.554		**.74<br><.001***	-.21<br>.226	-.07<br>.709	.14<br>.436	**-.31<br>.076†**	-.28<br>.113	**.34<br>.053†**	.02<br>.911	<.01<br>.980	**.30<br>.086†**
	RTV	**-.50<br>.003**	.22<br>.221		.17<br>.343	-.11<br>.524	.22<br>.209	-.18<br>.301	**-.53<br>.001**	.14<br>.435	-.03<br>.864	-.13<br>.480	.13<br>.471
	Commission errors	**-.41<br>.016***	-.01<br>.937	.23<br>.197		.19<br>.280	-.01<br>.952	.10<br>.587	**-.41<br>.016***	.07<br>.704	.17<br>.345	-.04<br>.832	-.20<br>.247
	Orienting-P1$_{diff}$ [a]	-.21<br>.229	-.22<br>.208	.12<br>.510	-.06<br>.731		-.18<br>.324	**.56<br><.001***	<-.01<br>.989	**.44<br>.009**	-.22<br>.205	.09<br>.600	.02<br>.906
	Orienting-P2$_{diff}$ [b]	.22<br>.202	.29<br>.097	-.05<br>.771	.08<br>.635	-.24<br>.164		-.25<br>.163	-.20<br>.265	-.10<br>.576	.16<br>.372	.05<br>.801	.16<br>.362
	Orienting-P3a$_{diff}$ [c]	.19<br>.271	-.26<br>.142	**-.46<br>.006**	-.08<br>.648	.29<br>.091	-.26<br>.141		**.31<br>.076†**	.01<br>.974	-.17<br>.342	.08<br>.643	-.10<br>.579
	Orienting-P3b$_{diff}$ [d]	.19<br>.274	**-.35<br>.040***	-.20<br>.253	-.22<br>.210	.08<br>.642	**-.44<br>.010***	.26<br>.143		.05<br>.802	.02<br>.925	.29<br>.101	**-.35<br>.042***
	Target-P1$_{diff}$ [e]	.21<br>.230	-.12<br>.487	-.04<br>.836	-.07<br>.698	-.13<br>.468	.24<br>.165	.01<br>.957	-.09<br>.598		-.13<br>.465	**.37<br>.029***	.01<br>.955
	Target-P2$_{diff}$ [f]	**.30<br>.097†**	-.26<br>.134	-.15<br>.384	-.10<br>.570	.01<br>.987	-.04<br>.833	-.11<br>.555	.29<br>.101	**.43<br>.011***		-.18<br>.296	-.12<br>.515
	Target-P3a$_{diff}$ [g]	.29<br>.101	**-.34<br>.052†**	-.17<br>.345	**-.35<br>.042***	.16<br>.370	.18<br>.317	-.05<br>.762	.009<br>.959	**.47<br>.005**	**.42<br>.013***		.19<br>.280
	Target-P3b$_{diff}$ [h]	.10<br>.574	-.11<br>.547	.01<br>.951	.05<br>.761	.05<br>.776	.26<br>.135	.03<br>.862	-.18<br>.308	.09<br>.601	.08<br>.646	**.65<br><.001***	

[a] Orienting-P1$_{diff}$ measured at right-occipital cluster for very preterm children, and left-occipital cluster for term children; [b] Orienting-P2$_{diff}$ measured at mid-frontal cluster for both groups; [c] Orienting-P3a$_{diff}$ measured at left-occipital cluster for both groups; [d] Orienting-P3b$_{diff}$ measured at mid-occipital cluster for both groups; [e] Target-P1$_{diff}$ measured at mid-parietal cluster for very preterm children and right-parietal cluster for term children; [f] Target-P2$_{diff}$ measured at mid-central cluster for both groups; [g] Target-P3a$_{diff}$ measured at mid-parietal clusters for very preterm children, right-parietal clusters for term children; [h] Target-P3b$_{diff}$ measured at mid-parietal clusters for both groups.

† p<.1; * p<.05; ** p<.01; ***p<.001; Blue shading reflects correlations between task performance and ERP metrics

Table S3: Correlations between task performance and ERPs on the motivating task variant

		Term											
		Hits	RT	RTV	Comm. errors	Orienting-P1$_{diff}$ [a]	Orienting-P2$_{diff}$ [b]	Orienting-P3a$_{diff}$ [c]	Orienting-P3b$_{diff}$ [d]	Target-P1$_{diff}$ [e]	Target-P2$_{diff}$ [f]	Target-P3a$_{diff}$ [g]	Target-P3b$_{diff}$ [g]
Very Preterm	Hits		**-.34<br>.047***	-.27<br>.119	**-.55<br><.001*****	-.17<br>.344	.07<br>.713	-.14<br>.414	.20<br>.256	.07<br>.694	.06<br>.722	.24<br>.172	-.01<br>.937
	RT	-.08<br>.652		**.47<br>.005****	**.55<br><.001*****	.15<br>.406	-.05<br>.765	.09<br>.605	.03<br>.873	-.08<br>.656	-.28<br>.106	-.20<br>.261	.04<br>.820
	RTV	**-.44<br>.009****	.18<br>.306		**.53<br>.001****	.12<br>.509	-.08<br>.650	.01<br>.941	**-.32<br>.065†**	-.04<br>.804	-.26<br>.144	-.24<br>.168	**-.31<br>.079†**
	Commission errors	**-.61<br><.001*****	-.17<br>.330	**.66<br><.001*****		**.40<br>.019***	**-.36<br>.037***	.28<br>.113	-.20<br>.257	-.12<br>.508	-.04<br>.811	**-.32<br>.063†**	-.27<br>.119
	Orienting-P1$_{diff}$ [a]	.10<br>.556	-.11<br>.547	.11<br>.534	.04<br>.908		**-.62<br><.001*****	**.78<br><.001*****	**.30<br>.085†**	<.01<br>.986	.22<br>.202	-.13<br>.466	-.07<br>.703
	Orienting-P2$_{diff}$ [b]	-.25<br>.156	.20<br>.256	.06<br>.734	.14<br>.441	**-.57<br><.001*****		**-.38<br>.028***	-.10<br>.580	-.15<br>.406	-.09<br>.595	.03<br>.881	-.10<br>.586
	Orienting-P3a$_{diff}$ [c]	**.34<br>.050†**	-.19<br>.293	-.04<br>.839	-.20<br>.255	**.37<br>.031***	**-.46<br>.007****		**.51<br>.002****	-.25<br>.159	**.36<br>.038***	-.05<br>.761	.01<br>950
	Orienting-P3b$_{diff}$ [d]	**.32<br>.062†**	-.15<br>.399	-.08<br>.643	-.15<br>.409	.04<br>.809	-.21<br>.238	**.75<br><.001*****		.05<br>.784	.09<br>.621	.09<br>.631	**.35<br>.043***
	Target-P1$_{diff}$ [e]	.14<br>.440	.13<br>.447	-.04<br>.836	-.07<br>.706	**.30<br>.085†**	-.05<br>.763	.29<br>.091	-.05<br>.770		**-.36<br>.034***	.21<br>.230	.14<br>.446
	Target-P2$_{diff}$ [f]	.15<br>.399	**-.39<br>.022***	**-.39<br>.023***	-.19<br>.286	-.29<br>.101	-.10<br>.576	-.06<br>.755	.06<br>.732	-.04<br>.819		.02<br>.889	-.22<br>.215
	Target-P3a$_{diff}$ [g]	-.01<br>.935	.12<br>.513	-.25<br>.153	-.08<br>.644	.01<br>.938	.21<br>.234	-.19<br>.280	-.22<br>.201	**.44<br>.010***	.18<br>.298		**.55<br><.001*****
	Target-P3b$_{diff}$ [g]	-.11<br>.540	.05<br>.798	-.04<br>.807	<.01<br>.996	**.30<br>.084†**	-.05<br>.791	-.10<br>.573	-.24<br>.167	.14<br>.422	-.16<br>.367	**.64<br><.001*****	

[a] Orienting-P1$_{diff}$ measured at right-occipital cluster for very preterm children, and left-occipital cluster for term children; [b] Orienting-P2$_{diff}$ measured at mid-frontal cluster for very preterm children, left-frontal cluster for term children; [c] Orienting-P3a$_{diff}$ measured at left-occipital cluster for very preterm children, right-occipital cluster for term-born children; [d] Orienting-P3b$_{diff}$ measured at mid-occipital cluster for very preterm children and right-occipital cluster for term children; [e] Target-P1$_{diff}$ measured at left-occipital cluster for both groups; [f] Target-P2$_{diff}$ measured at mid-central cluster for both groups; [g] Target-P3a$_{dif}$ and Target-P3b$_{diff}$ measured at mid-parietal clusters for both groups

† p<.1; * p<.05; ** p<.01; ***p<.001; Blue shading reflects correlations between task performance and ERP metrics
